# An Ancient Fingerprint Indicates the Common Ancestry of Rossmann-Fold Enzymes Utilizing Different Ribose-Based Cofactors

**DOI:** 10.1371/journal.pbio.1002396

**Published:** 2016-03-03

**Authors:** Paola Laurino, Ágnes Tóth-Petróczy, Rubén Meana-Pañeda, Wei Lin, Donald G. Truhlar, Dan S. Tawfik

**Affiliations:** 1 Department of Biological Chemistry, Weizmann Institute of Science, Rehovot, Israel; 2 Department of Chemistry, Chemical Theory Center, and Supercomputing Institute, University of Minnesota, Minneapolis, Minnesota, United States of America; University College London, UNITED KINGDOM

## Abstract

Nucleoside-based cofactors are presumed to have preceded proteins. The Rossmann fold is one of the most ancient and functionally diverse protein folds, and most Rossmann enzymes utilize nucleoside-based cofactors. We analyzed an omnipresent Rossmann ribose-binding interaction: a carboxylate side chain at the tip of the second β-strand (β2-Asp/Glu). We identified a canonical motif, defined by the β2-topology and unique geometry. The latter relates to the interaction being bidentate (both ribose hydroxyls interacting with the carboxylate oxygens), to the angle between the carboxylate and the ribose, and to the ribose’s ring configuration. We found that this canonical motif exhibits hallmarks of divergence rather than convergence. It is uniquely found in Rossmann enzymes that use different cofactors, primarily SAM (S-adenosyl methionine), NAD (nicotinamide adenine dinucleotide), and FAD (flavin adenine dinucleotide). Ribose-carboxylate bidentate interactions in other folds are not only rare but also have a different topology and geometry. We further show that the canonical geometry is not dictated by a physical constraint—geometries found in noncanonical interactions have similar calculated bond energies. Overall, these data indicate the divergence of several major Rossmann-fold enzyme classes, with different cofactors and catalytic chemistries, from a common pre-LUCA (last universal common ancestor) ancestor that possessed the β2-Asp/Glu motif.

## Abstract 2

The widely distributed Rossmann-fold enzymes share a highly conserved geometry of their ribose binding motif; this geometry is very rarely found in other folds and represents a relic of a common ancestral enzyme.

## Author Summary

Common descent is the hallmark of Darwinian evolution. Homology of biological traits, and particularly of protein sequences and structures, serves as an indication for divergence from a common ancestor and a means of assigning phylogenetic relationships. However, because of shared functional demands and chemical-physical constraints, proteins that evolved independently of one another often converge on very similar molecular traits, including structure and sequence. We tested the widely accepted hypothesis of common ancestry of several major enzyme classes, comprising hundreds of different families and using different cofactors and catalytic chemistries. Although they share the same overall architecture—the Rossmann fold—these enzymes show no significant sequence homology across different classes. We describe an analysis based on the omnipresence of a single residue across these classes: an acidic aspartate or glutamate residue that binds ribose, the common denominator of the different cofactors used by these enzymes. We show that Rossmann enzymes possess a unique interaction geometry that represents a fingerprint of common ancestry rather than an outcome of molecular constraint. We thus provide the first systematic test of divergence versus convergence of a highly abundant protein motif and assign common descent in one of the most ancient and functionally diverse protein folds.

## Introduction

Nucleoside-based cofactors are widely abundant and are likely to have appeared well before proteins [1–3]. The early protein forms may have therefore evolved to bind and function with nucleoside-based cofactors [4]. However, tracing motifs that relate to the earliest stages of protein-cofactor evolution is a challenge [5]. Omnipresent cofactor-binding motifs, such as the P-loop (phosphate-binding loop or Walker A motif), are considered fingerprints of the earliest precursors of modern proteins [5]. However, in general, abundance of a trait per se (in terms of number of species and their distribution in the tree of life) is not sufficient to indicate common ancestry, as convergence of sequence and structure is a feasible alternative. The more minimal a motif is in terms of the number of amino acids, the more likely it is to be the outcome of convergent evolution—namely, to have evolved independently, along separate lineages, yet ended up with the same molecular solution [6]. In fact, there is ample evidence for convergence, both of structural architectures (folds) and of binding and catalytic motifs. Folds such as β-propellers, for example, have emerged in parallel many times [7–10]. Artificial proteins belonging to the most ancient folds are computationally designed with sequences that bear no relation to natural proteins [8,9]. Omnipresent catalytic motifs such as the Asp/Glu dyads of glycosyl hydrolase and transferases are seen in >50 different folds [11] and with no significant sequence homology beyond the dyad itself. Such motifs have probably emerged independently, and their conserved geometry is due to physicochemical constraints dictated by a shared function. In fact, when it comes to binding and catalytic motifs, convergence is probably as dominant as divergence [12]. Overall, differentiating divergent from convergent evolution remains a crucial, largely unresolved dilemma in evolutionary biology in general and in protein evolution in particular [13–16].

**Fig 1:**
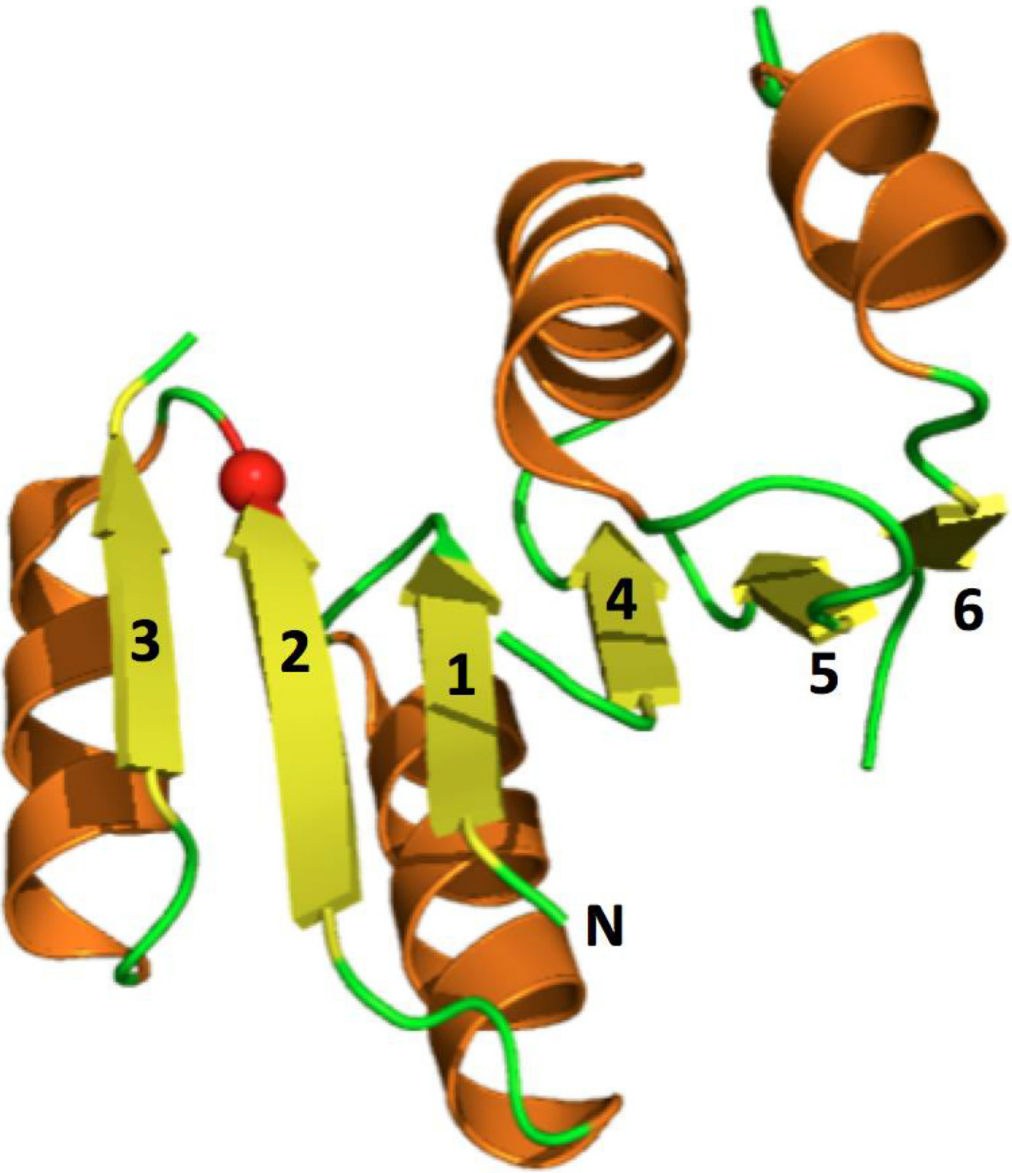
Schematic representations of the architecture of the Rossmann fold. The Rossmann is a β/α fold, namely a consecutive repeat of motifs comprising a β-strand (in yellow), a connecting loop (green), and α-helix (orange). The β-strands form a core β-sheet with the strands in the distinct order of 3-2-1–4-5-6. In effect, the Rossmann fold comprises two consecutive repeats, each comprising three β-strands (3-2-1 and 4-5-6), and two connecting α-helices. Shown in a red sphere is the ribose-binding Asp/Glu residue analyzed here, which resides at the tip of the second β-strand. Note that the Rossmann fold is usually addressed in the wider context, including Rossmann-like, or Rossmanoids, in which the sixth strand is missing, or is modified with additional secondary structural elements (e.g., methyltransferases).

Our study focuses on the Rossmann fold. By virtue of catalyzing >300 different enzymatic reactions [17], the Rossmann fold is one of the most widely occurring protein folds [18–21] and is accordingly well represented in the presumed set of proteins that existed in the last universal common ancestor (LUCA) [20,22,23]. Belonging to the general class of β/α proteins, the Rossmann fold comprises two tandem repeats. Each repeat comprises three consecutive strands forming a parallel pleated sheet and two connecting α-helices [24–26]. The strand order along the core β-sheet is 3-2-1–4-5-6, although modifications of the last strand are often seen (Fig 1). Rossmann-fold enzyme families are also characterized by their use of cofactors [20,27,28] and in particular of nucleoside-containing cofactors that were present in the presumed “RNA world,” prior to the emergence of proteins [1,2]. Rossmann-fold enzymes therefore comprise a clear example of the evolutionary link between cofactors and their utilizing enzymes. Indications for pre-LUCA evolutionary links in the Rossmann fold have been noted that relate to nucleoside binding and the shared fold [19,29]. Shared nucleoside binding motifs have also been described upon the identification of the Rossmann fold and at later stages (e.g., [6,30–39]). Specifically, nicotinamide adenine dinucleotide (NAD)- and flavin adenine dinucleotide (FAD)-utilizing enzymes share a Gly-rich loop that resides between H1 and β1 and interacts with the cofactors’ phosphate moieties [19,40,41], and the hydroxyls of the cofactors’ ribose moiety typically interact with a Glu/Asp at the tip of β2 (β2-Asp/Glu; Fig 1) [42,43]. Sequence homology can obviously be detected between NAD and nicotinamide adenine dinucleotide phosphate (NADP) enzymes and may span over to FAD enzymes, specifically in relation to the above two motifs [44,45]. However, the sequence homology with other Rossmann classes such as S-adenosyl methionine (SAM)-dependent methyltransferases is much less clear [36,44]. The ribose-binding Glu/Asp at the tip of β2 has also been detected in methyltransferases [42,43]. However, the Gly-rich motif is not apparent in SAM-utilizing Rossmann enzymes, possibly because SAM does not contain phosphate groups. Consequently, some sequence-based classifiers, including those using sensitive homology detectors such as CATH (Class Architecture Topology Homologous superfamilies), define these classes as separate superfamilies [46]. However, based amongst other considerations on the shared β2-Asp/Glu motif, other classifiers such as ECOD (Evolutionary Classification of Protein Domains) [30] or Interpro [47] classify all three classes (NAD(P), FAD, and SAM-dependent Rossmann enzymes) in the same homology group [31,32,35,38,39].

Overall, a common fold [20] and the shared binding motif (the ribose β2-Asp/Glu interaction) are highly suggestive of a common Rossmann ancestor and specifically of common ancestry of NAD-, FAD-, and SAM-utilizing enzymes [30,34,38]. Indeed, these three classes (and a few additional ones addressed below) are all present in the presumed LUCA [48,49]. However, so far, there has been no attempt, to our knowledge, to examine whether these shared features are indeed a hallmark of common descent [39]. Such a systematic analysis is crucial in view of convergence being common and especially because the shared binding motif comprises a single residue.

## Results

### The Bidentate Ribose-Carboxylate Interaction

**Fig 2:**
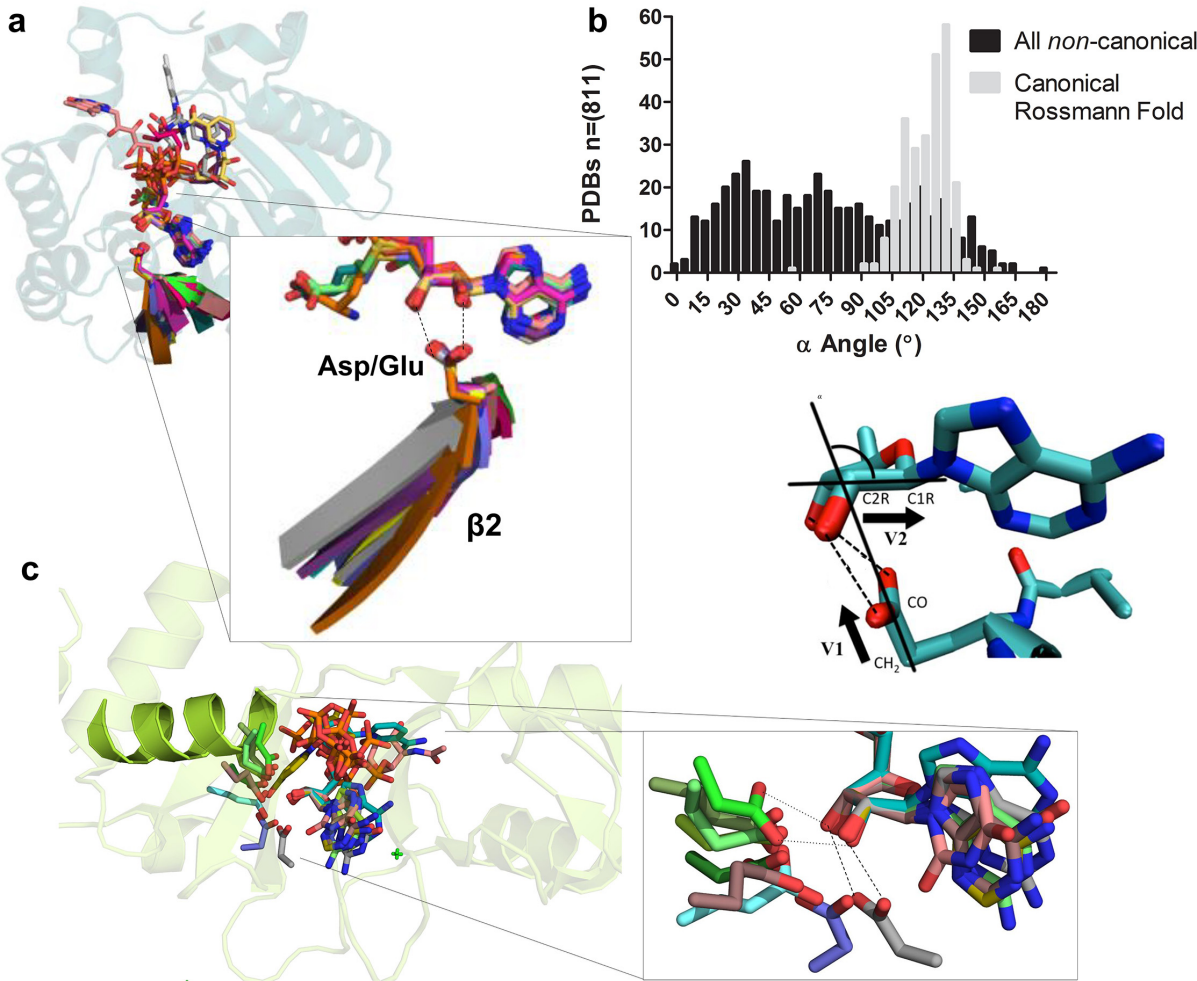
The geometrical and topological features of the canonical Rossmann β2-Asp/Glu motif. (A) Representative carboxylate-ribose bidentate interactions in Rossmann-fold enzymes. Structures were superpositioned by the ribose moiety of their cofactors. One complete backbone is shown (in cartoon), whilst for others, shown are the bound cofactor, the second β-strand (β2), and the interacting Glu or Asp. PDB (Protein Data Bank) IDs and corresponding cofactors: 1JG2, ADN; 3GVI, ADP; 2HMU, ATP; 2XXB, AMP; 1BWC, FAD; 1V5E, FAD; 1EG2, MTA; 2A14, 2PBF, 2AVD (complete structure), SAM; 2GR2, FAD; 1AHH, NAD; 1GEG, NAD; 1GZ6, NAI. (B) The distribution of the interaction angle (α) in structures of proteins with a ribose bound to an Asp/Glu via a bidentate interaction. α is defined by two vectors: v_1_, going through the CH_2_-COO^-^ carbons of the interacting Asp/Glu side chain, and v_2_, going through the C1-C2 carbons of the ribose ring. Gray bars represent the angles in all Rossmann structures with the canonical motif (*n* = 263). Black bars represent the angles of all the noncanonical bidentate interactions found in both Rossmann and non-Rossmanns enzymes. The PDB Rossmann structures with canonical and noncanonical interaction and their α angles are listed in S1 Data. (C) Representative noncanonical bidentate interactions in non-Rossmann enzymes. PDBs and the corresponding cofactor: 1HO5, ADN; 2J9L (complete structure; the helix carrying the ribose-binding Asp727 is highlighted), ATP; 3S2U, UD1; 1K9Y, AMP; 2ATV, GDP; 1SIW, GDP; 3TE5, NAI; 1I7L, ATP; 4B45, GSP. These structures are shown individually in S6 Fig.

We were initially interested in engineering the SAM-binding site of DNA methyltransferases—a Rossmann-fold enzyme superfamily. Our attention was focused on the adenosine group that appears in nearly all of the key enzymatic cofactors. In this context, we were searching for a highly conserved interaction that is critical to adenosine binding and could be modified. However, our analysis indicated that none of the residues that interact with the adenine ring are conserved in all DNA methyltransferases. In contrast, we observed that a Glu residue that interacts with the ribose is entirely conserved. We first observed that the carboxylate-ribose interaction is completely conserved in SAM-dependent methyltransferases, including DNA, RNA, protein, and small molecule methyltransferases. We realized that conservation does not simply concern an active-site Asp/Glu that interacts with SAM [42,43] but primarily relates to a bidentate interaction with the ribose’s 2ʹ and 3ʹ hydroxyls with an unusually narrow distribution of H-bond distances and angles. Distinctly, the interacting Asp/Glu is at the tip of the Rossmann’s second beta strand (β2) (Fig 2A; S1 Fig and S2 Fig). Further, although the β2-Asp/Glu was described as a characteristic of Rossmann NAD dehydrogenases [44], its bidentate nature has not been described as such.

A wider examination that further included NAD- and FAD-dependent oxidreductases was performed (see Methods and S3 Fig). This analysis confirmed that, as suggested earlier [40,41,50], the ribose-interacting Asp/Glu is also widely spread in these two enzyme classes. However, to our knowledge, the prevalence of this Asp/Glu interaction across NAD/FAD oxidoreductases, as well as SAM-dependent methyltransferases, and the geometrical conservation of the bidentate interaction with the bound ribose have not been previously noted. We therefore defined a new canonical Rossmann motif based on four criteria: (*i*) a tight, bidentate interaction exists between a carboxylate side chain and the ribose’s 2ʹ and 3ʹ-hydroxyls; (*ii*) the ribose’s furanose ring conformation is in an envelope form, mainly the E_1_ and ^2^E conformations (S4 Fig: see also S1 Text); (*iii*) the angle the ribose and the interacting carboxylate (hereafter the ribose–carboxylate angle α; defined in Fig 2B) is 90°–140°; and (*iv*) the interacting Glu/Asp is located at the tip of the β2 strand of the Rossmann fold (Fig 2A).

### The Canonical Rossmann Interaction

A systematic analysis identified the above motif features as being unique to the Rossmann fold. All nonredundant PDB structures containing ribose ligands were downloaded (Table 1; *n* = 2,949; S5 Fig). Of these, ~30% were found to have a carboxylate side chain that is within interacting distance (≤3.4 Å) of both the 2ʹ and 3ʹ hydroxyls of the ribose (*n* = 811). These structures were then categorized by the angle α (Fig 2B). The secondary structural element to which the interacting Glu/Asp residue belongs was also classified, as well as the fold (using Structural Classification of Proteins [SCOP] and/or CATH annotations). This analysis indicated that the canonical bidentate interaction underlies enzyme families and superfamilies that possess a Rossmann fold. Specifically, the canonical interaction was found in 54% of the structures classified as a Rossmann fold (Table 1). These structures were manually examined, and the order of their β-strands was found to fit the Rossmann-fold topology. Further, ≥96% of the examined Rossmann enzymes have their ribose rings in the ^2^E or E_1_ configuration (discussed below). Only 8% of the structures belonging to the Rossmann fold possessed noncanonical interactions—namely, bidentate interactions with α < 90° or > 140° and/or with the interacting Glu/Asp not being located at the tip of a β strand. Conversely, in enzymes belonging to non-Rossmann folds, monodentate or no Asp/Glu interactions are the rule (91%). Further, when bidentate interactions are present in non-Rossmann proteins, they almost never meet the canonical criteria, namely the canonical angle and the interacting Glu/Asp being at the tip of a β-strand. Indeed, amongst non-Rossmann enzymes, only 1.7% exhibit bidentate interactions that meet the canonical criteria versus 6% that exhibit bidentate interactions that do not meet the canonical criteria; Fig 2A–2C, S6 Fig).

**Table 1:** The occurrence of carboxylate-ribose interactions in all known protein structures with ribose-containing ligands.

	Bidentate Asp/Glu Interactions		Other Interactions	
	Canonical	Noncanonical	Monodentate Asp/Glu Interactions	No Glu/Asp Interaction
Rossmann fold (***n* = 484**)	263 (54%)	38 (8%)	66 (14%)	117 (24%)
P-loop nucleoside triphosphatases (NTPases) (***n* = 210**)	0 (0%)	2 (1%)	17 (8%)	191 (91%)
Non-Rossmann folds (***n* = 901**)^1^	27 15(1.7%)^1^	52 (6%)	179 (20%)	643 (71%)
No assigned fold (***n* = 1,354**)^2^	279 (20%)	150 (11%)	249 (18.4%)	676 (50%)
Total (***n* = 2,949**)	578	233	511	1,627
Methyltransferases^3^ (***n* = 55**)	50 (91%)	n.d.	0 (0%)	5 (9%)^4^
NAD/FAD-utilizing enzymes^3^ (***n* = 315**)	228 (73%)	n.d.	22 (7%)	65 (20%)

The analysis includes all deposited nonredundant PDB structures circa July 2014, with <2.5 Å resolution and with a ligand containing a ribose with unmodified 2ʹ and 3ʹ hydroxyls (*n* = 2,739). Fold categories are defined in the Methods.

^1^ Initially, 27 non-Rossmann PDB structures were identified by the computational search as having a canonical motif. These were manually examined, and consequently eight structures that are clearly Rossmann or Rossmannoids (1DJN, 1GTE, 1PS9, 1I8T, 2C31, 2E5W, 3C6K, and 2DHP) were excluded. It appears that their CATH/SCOP non-Rossmann annotations were derived primarily from additional domains in these structures.

^2^ Structures for which neither a SCOP nor a CATH category is specified in the PDB (SCOP v.1.75 and CATH_v3.5.0, version date: 20.09.2013 used for this analysis).

^3^ Superfamily specific statistics for methyltransferases (SCOP families c.66.1 structures bound to SAM) and of NAD/FAD dehydrogenases (SCOP families c.2.1 and c.3.1.5). n.d. = not determined.

^4^ A profound change in the SAM-binding site was observed in these five structures, whereby a long loop extending from β2 interacts with the ribose hydroxyls.

One notable example showing how unique the canonical motif is to the Rossmann fold is the P-loop nucleoside-triphosphatase (NTPase) fold (CATH annotation 3.40.50.300; SCOP superfamily c.37.1, P-loop containing nucleoside triphosphate hydrolase). This fold also belongs to the class of β/α proteins. Overall, its topology is highly similar to the Rossmann fold, except that the order of strands within its core β-sheet is 2-3-1–4-5-6. Thus, the location of β2, where the canonical Rossmann Asp/Glu ribose-binding residue appears (Fig 1), is shifted relative to the Rossmann topology. We found that none of the structures belonging to the P-loop NTPases superfamily (CATH Family 3.40.50.300; *n* = 210) contains the canonical carboxylate-ribose interaction. Further, as discussed below, the mode of nucleoside binding in P-loop NTPases differs fundamentally from the one observed in the Rossmann fold.

### The Canonical Motif Is a Rossmann-Fold Identifier

Nearly half of the structures (279/578) in our original dataset were found to have the canonical carboxylate-ribose interaction but had no SCOP or CATH category (Table 1). We manually examined all 279 structures and found that 271 of these structures have a Rossmann, or Rossmann-like, topology, as defined above, and with the interacting Glu/Asp located at the tip of β2 (S5 and S6 Tables, S7 Fig). In fact, 108 out of the 279 structures that were not annotated in the CATH version v3.5.0 used to make our dataset are annotated in the current version (v.4.0.0; in which the number of annotated domains is larger by 36%). This “blind test” indicates that the applied criteria are sufficient not only to identify the canonical motif in Rossmann enzymes but also to rigorously identify a Rossmann enzyme merely by the existence of this canonical motif.

### The Canonical Motif in NAD Enzymes Is Adenosine Specific

**Fig 3:**
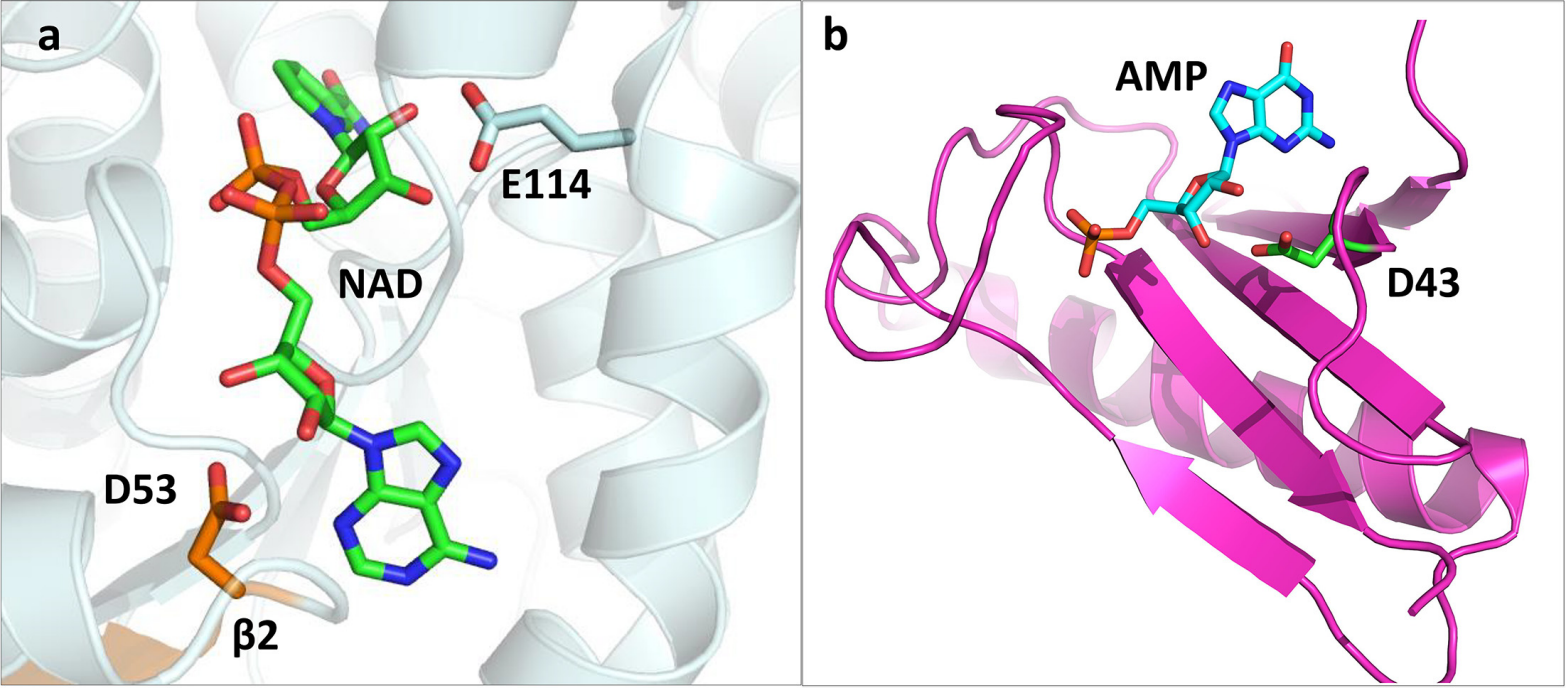
Representative noncanonical bidentate interactions in Rossmann and non-Rossmann enzymes. (A) Zoom-in view of the structure of L-3-hydroxyacyl-CoA dehydrogenase belonging to the Rossmann fold (PDB 1F17). The cofactor, NAD (in green sticks), has its adenosine ribose interacting with D53 located at the tip of β2, with a canonical angle (α = 120°). A second bidentate interaction is observed that is not observed in other Rossmann NAD dehydrogenases, between the nicotinamide ribose and E114 located on H4 and with a noncanonical angle (α = 16°). (B) Zoom-in view of the HIT protein (HINT, histidine triad), a non-Rossmann enzyme in which a carboxylate-ribose bidentate is observed (PDB 3RHN). The cofactor, adenosine monophosphate (AMP), is in cyan sticks. The interacting D43 is at the tip of a β-strand and with a canonical angle (α = 116°). Note, that the β-strand is part of an antiparallel sheet, in oppose to the parallel β-sheet that comprises the Rossmann’s core (Fig 1).

NAD-utilizing enzymes provide another indication for divergence from a common adenosine-binding ancestor. The cofactor NAD contains two riboses, one attached to adenosine and the other to nicotinamide. However, in the 259 available structures of NAD-dependent enzymes, only bidentate carboxylate-ribose interaction was found with the ribose. Among the NAD enzymes annotated as Rossmann, 145 structures out of 155 fit the canonical criteria with respect to the interaction with the adenosine’s ribose (S7 Table). Only four structures possess an additional bidentate interaction with NAD’s nicotinamide ribose. Of these four, two are annotated as Rossmann folds. Both these structures have one canonical interaction at the tip of β2 binding the adenosine ribose, as do the 145 other NAD Rossmann-fold enzymes. The nicotinamide riboses, however, interact with Glu residues located not at the tip of β2, and these bidentate interactions exhibit noncanonical geometries (Fig 3A and S8 Fig). The variability of the ribose-carboxylate angles and topology (Asp/Glu locations other than β2) and the sporadic presence (4/155 indicating appearance in recently evolved lineages) are all consistent with emergence by convergence. In contrast, the prevalence (145/155) and conservation of both geometry and topology of the interaction with the adenosine’s ribose most likely indicates divergence from a primordial ancestor of the Rossmann fold.

### Experimental Examination of the Canonical Interaction

**Fig 4:**
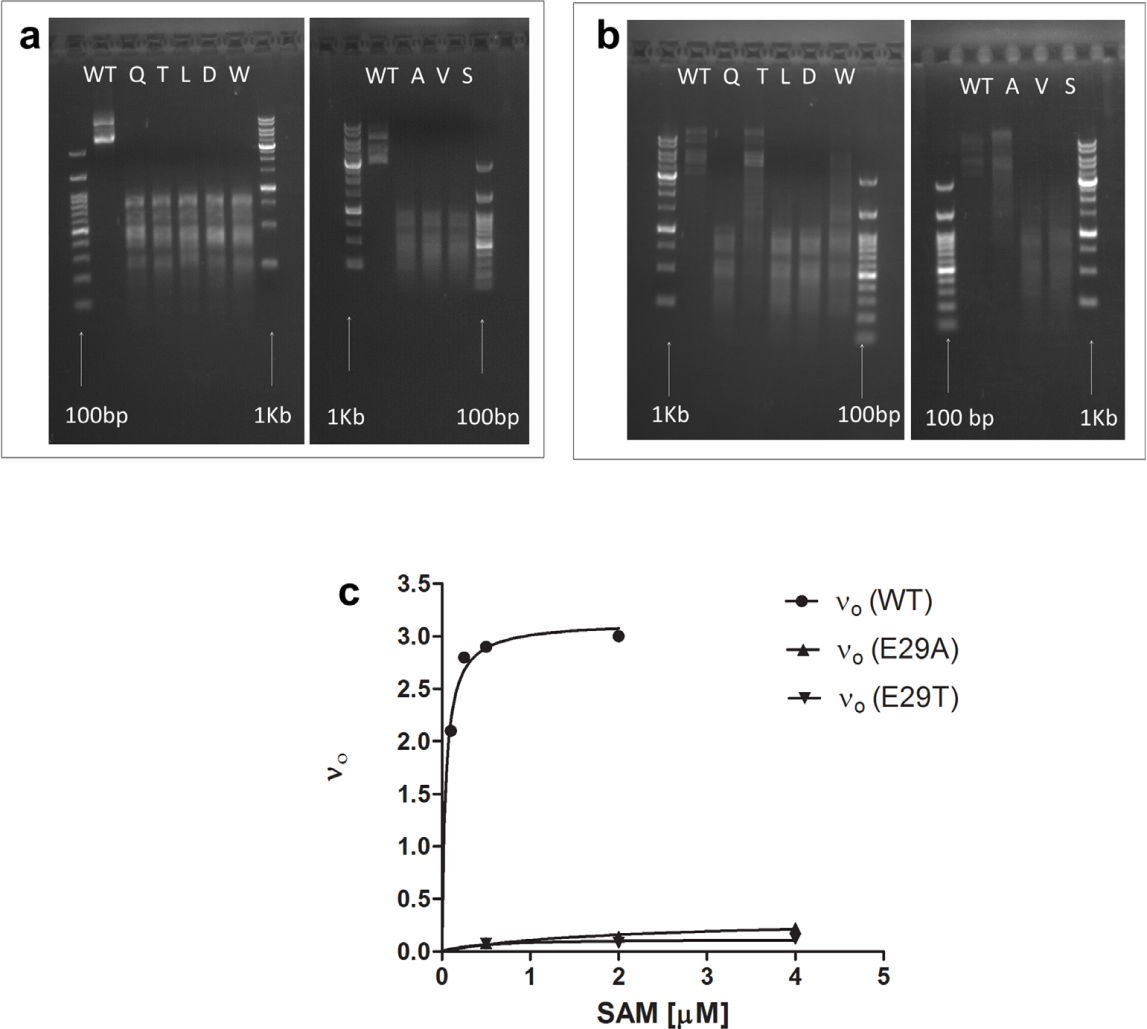
Experimental examination of the carboxylate-ribose bidentate interaction in DNA methyltransferase M.*Hae*III. (A) The methylation activity by plasmid protection. Plasmids encoding wild-type M.*Hae*III and its E29 mutants were transformed to *Escherichia coli*. Following growth and basal expression of the methyltransferase (no inducer), plasmid DNA was extracted and treated with the restriction enzyme, HaeIII. As can be seen, none of the E29 mutants were capable of methylating its plasmid, as indicated by complete digestion into fragments. The arrows identify the DNA ladders. (B) The assay was repeated with the wild type and the mutants being overexpressed (with inducer). Under these conditions, the assay sensitivity is very high, and variants whose expression or activity is well under 100-fold compared to wild type show 100% protection [53]. (C) Michaelis-Menten curves for wild-type M.*Hae*III (WT) and of the E29 mutants exhibiting detectable activity. Time-dependent in vitro methylation assays were performed as described, and initial reaction rates (*v*_*0*_) were extrapolated from the linear phase in the time-dependent courses of the reaction (raw data are available in S2 Data). Experiments were carried out at 37°C, with WT M.*Hae*III at 0.1 μM and the E29 mutants at 8 μM.

A motif that has been retained for ≥3.7 billion y of evolution is likely to be functionally important. Indeed, the contribution of the Glu/Asp interaction in NAD- and FAD-utilizing enzymes is widely recorded (published data listed in S8 Table) [51,52]. However, we could not find reports describing the experimental examination of its role in SAM-utilizing enzymes. To this end, we examined a typical bacterial mC5 DNA methyltransferase, M.*Hae*III, in which Glu29 interacts with the SAM cofactor with the canonical motif geometry (Fig 4), as do nearly all other Rossmann methyltransferases (Table 1). Methylation activity was completely lost upon replacement of Glu29, including conservative replacements such as Gln, or Asp, and dropped by up to 450-fold in terms of *k*_*cat*_ */K*_*M*_ in the Glu29Thr and Ala mutants (Fig 4, S8 Table). Overall, it appears that the canonical bidentate interaction have an important contribution to cofactor binding in the three classes of Rossmann enzymes in which it prevails, namely in NAD-, FAD-, and SAM-utilizing enzymes. However, the effects of mutations seemed to differ; for example, in glyceraldehyde-3-phosphate dehydrogenase (GAPDH) (NAD dependent) and sarcosine oxidase (FAD dependent), the conservative D to E mutations reduced *k*_*cat*_ */K*_*M*_ by ≤10-fold, whereas in M.*Hae*III (SAM dependent), activity was completely lost. Thus, in all three enzymes, relatively conservative exchanges such as D to A or D to N resulted in up to 90-fold losses, yet the loss of activity observed for the SAM-dependent M.*Hae*III was generally higher. The contribution of the bidentate interaction to SAM binding is probably higher than in the case of NAD and FAD because in the latter two, the Asp/Glu bidentate interaction is further away from the reaction center.

### The Canonical Geometry: A Local Optimum but Not the Only One

Is the highly conserved geometry of the Rossmann bidentate motif the outcome of chance or of necessity [54]? Namely, does the canonical geometry comprise the most optimal mode of ribose binding, or is it just one out of several options? Evolution of the Rossmann fold and cofactor binding implies that a single solution was selected at the ancestral stage, presumably owing at least in part to its favorable binding energy, and has been conserved ever since. Indeed, a scenario of divergence typically follows from the existence of several possible solutions; in particular, divergence of the bidentate carboxylate interaction geometries would seem to imply that there are multiple such geometries of similar energy. Convergence, on the other hand, is compatible with a scenario whereby the bidentate interaction geometry seen in existing proteins is the only optimal one or even the only possible one.

We can illustrate the above line of reasoning by considering the dihedral angles (ω) of the peptide bonds in proteins. The distribution of ω along >200,000 peptide bonds in known protein structures is narrow, with a clear maximum at planarity (>97% of bonds within ω = 180 ± 10°). This distribution corresponds to a single optimum value of 180° [55]. The planarity of the peptide bond therefore relates to a physical constraint that dictates all protein structures, rather than to a trait that diverged from the very first peptide. Another example mentioned in the introduction is the Asp/Glu dyads seen in glycosydases of many different folds, whereby the intercarboxylate distances are highly conserved within two categories of retaining glycosidases (5.5 Å) and inverting ones (10 Å) [11].

**Fig 5:**
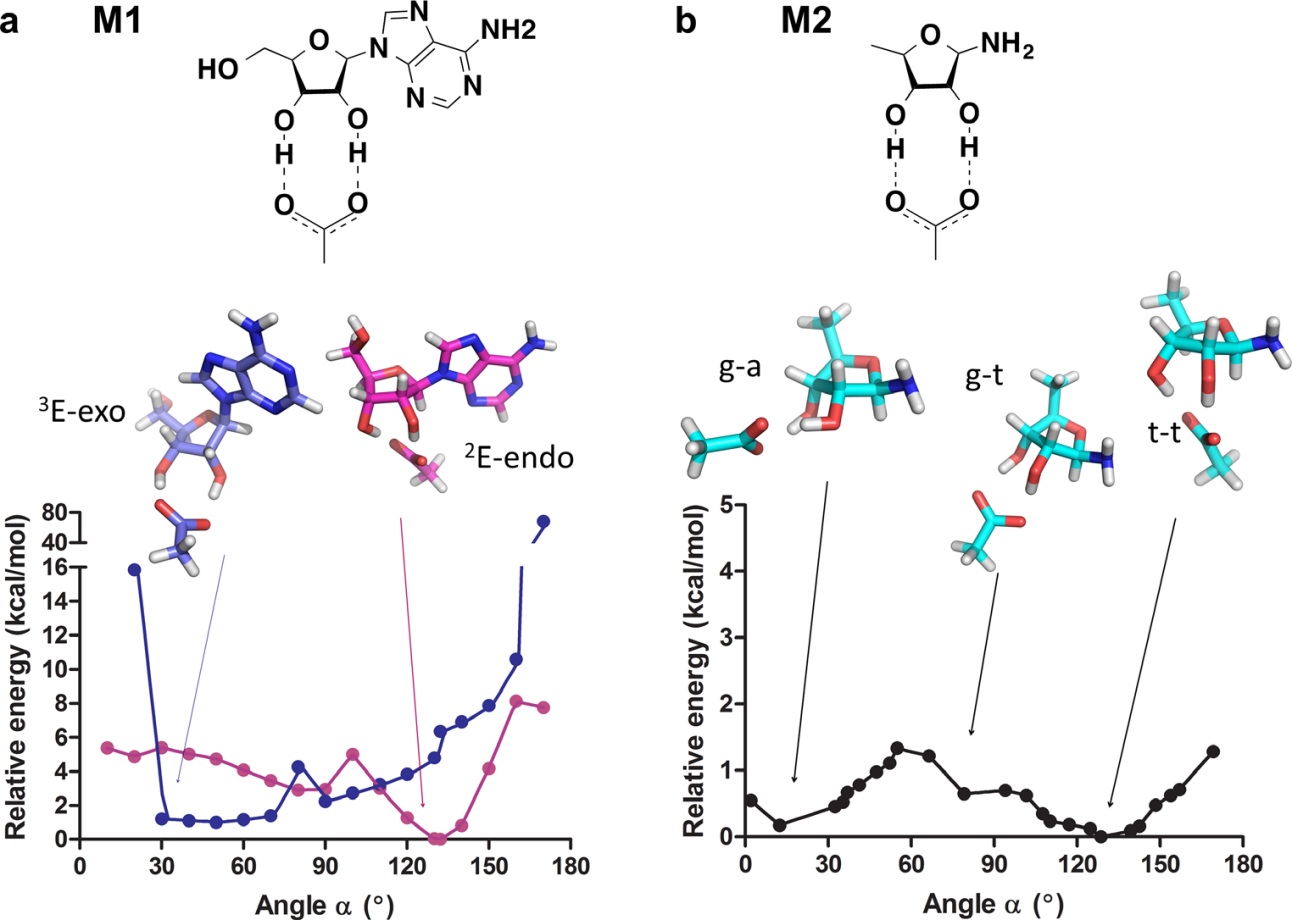
The potential energy of the bidentate ribose-carboxylate interaction as a function of the interaction angle. The energies, in kcal/mol, are plotted as a function of the carboxylate-ribose angle α and are relative to the lowest identified energy for each of the models. (A) Structure and energy profile for model M1 indicating the optimized lowest-energy structures, ^2^E-endo (in magenta) and ^3^E-exo (in blue). (B) Structure and energy profile for model M2, in which g-a, g-t and t-t are fully optimized structures. Note that the energy plots for the two models are drawn on two different scales (data available in S3 Data).

The favorable contribution of the bidentate carboxylate interaction to binding of vicinal-diols (as are the 2ʹ, 3ʹ hydroxyls of ribose) was indicated in small-molecule structures (S9 Fig) and by quantum mechanical calculations [56]. In the present work, we carried out new calculations to examine how energetically favorable is the geometry of the canonical interaction, and specifically how the energy of this interaction changes with the ribose-carboxylate angle (α) and ribose ring configuration. We performed quantum mechanical calculations designed to produce energy profiles of the different furanose configurations of ribose and of the ribose-carboxylate interaction angle (α) [57]. For this purpose, density functional theory electronic structure calculations with the Solvation Model based on Density (SMD) solvation model were used to study the ribose-carboxylate interaction in model systems in which the structures were energy minimized as a function of the ribose-carboxylate angle α (Fig 5; the energy calculations are described in detail in the S1 Text). The quantum mechanical calculations were performed on two models systems, M1 and M2, defined in Fig 5. After conformational searches, we identified the lowest-energy structures of model M2 (dubbed g-a, g-t, and t-t) and those for M1 (dubbed ^2^E-endo and ^3^E-exo). The lowest-energy structure obtained for M1 is ^2^E-endo, and for M2, it is t-t. Both ^2^E-endo and t-t exhibit a similar endo conformation, with respective α values of 132° and 129° and a similar envelope form for the ribose ring (^2^E for ^2^E-endo and E_1_ for t-t). The relative energy was accordingly plotted against the angle α (Fig 5A for model M1 and Fig 5B for model M2), indicating the lowest-energy structure for each value of α. These plots show that the bidentate interaction presents an angle optimum of ~130°. This optimum clearly overlaps the canonical Rossmann angle (Fig 2B). Further, the vast majority of Rossmann enzymes possess a ribose ring in a ^2^E or E_1_ configuration (96% of 263 PDB structures analyzed; see S1 Text) and an endo conformation (100% of 263 structures; see S1 Text), thus matching their modeled counterparts, ^2^E-endo and t-t.

However, beyond the canonical optimum, the potential energy surface for the carboxylate-bidentate interaction is relatively flat, with several minima. The only angles that appear to be highly disfavored are the edges, i.e., close to 0° and 180°, and these regions are also unoccupied in natural proteins (Fig 2B). Energy minima corresponding to the ^3^E-exo configuration for M1, and the g-a configuration for M2, are seen in α range of 10°–37° (Fig 5). According to our calculations, the endo configuration is more stable than the exo, by about 1 kcal/mol for model M1 and by only 0.1 kcal/mol for model M2. These differences are relatively small—an energy difference of 0.55 kcal/mol (the average difference for M1 and M2) corresponds to ~2.5-fold difference in affinity. For comparison, as indicated by the effects of mutations of the canonical Asp/Glu, the contribution of this interaction in Rossmann enzymes of different classes differs by well over 10-fold (see the above section and S8 Table).

The model structures that correspond to the alternative energy minima are seen in typical noncanonical interactions (Fig 2C, carboxyl side chains in variable greens). One characteristic example can be seen in Fig 3A, with the angles of the noncanonical interactions being 16°, far off the canonical range (90°–140°) and within the second predicted minimum (Fig 5). This alternative minimum corresponds to an exo disposition and has the ribose ring in the ^3^E for ^3^E-exo and in ^2^E for g-t. This mode is clearly seen in enzyme structures with the interaction angle in the range of 14° to 43° (Fig 2B and Fig 3), whereby the interaction corresponds to an exo configuration and the furanose conformation of the ribose is scattered among several possibilities (see S1 Text). Another example is human phosphoglyceraldehyde kinase where Glu344, located at the tip of β4, not β2, interacts with the ADP ribose in a bidentate manner, with the angle being 57° (S10 Fig).

Overall, the computations indicate that the canonical interaction is an intrinsically favorable mode for binding of ribose. It also corresponds to a furanose ring configuration that is the most energetically favored irrespective of the protein binding pocket and additional interactions, e.g., with the nucleoside’s base. However, the canonical interaction is only one out of at least two, if not more, favorable modes of bonding. Indeed, a wide distribution of interaction angles (Fig 2B) is seen in non-Rossmann ribose-binding proteins and predominantly in noncanonical interactions in Rossmann enzymes.

## Discussion

### Convergence or Divergence?

The utility of the carboxylate-ribose bidentate interaction, and its appearance in numerous protein families belonging to different folds and binding different cofactors, suggest that it arose independently, i.e., by convergent evolution. This is not surprising in view of the simplicity of this motif—a single carboxylate side chain aligned against the ribose hydroxyls. However, the statistics of occurrence clearly support the hypothesis of divergence. The canonical interaction is >30 times more frequent in Rossmann enzymes (54%) compared to non-Rossmann ones (1.7%). In contrast, the occurrence of noncanonical bidentate interactions in Rossmann and non-Rossmann proteins is nearly identical (8% and 6%, respectively; Table 1). Thus, whilst convergence to the canonical geometry and/or topology did occur, as exemplified in Fig 3B, its frequency of occurrence is not only lower but is also independent of the fold. The distinct features of convergence are apparent, including within Rossmann enzymes.

The distinct geometry of this motif in Rossmann enzymes may also provide a new means for automated classifications, as indicated by our manual examination of the structures with no CATH or SCOP annotations. The presence of an Asp/Glu at the loop connecting the second β-strand and the following helix is insufficient to distinguish between Rossmann from non-Rossmann enzymes (as previously noted [37,39] and also indicated by our data). However, when the carboxylate-ribose angle criterion is added, prediction accuracy increases to 97% (the false positive rate is 8/279).

The ancient origins of the ribose–(Asp/Glu-β2) motif and the claim for divergent evolution are also supported by the role of this motif in the switch of cofactor specificity of dehydrogenases. NADP-dependent dehydrogenases seem to have diverged from NAD-dependent enzymes [58], probably along multiple lineages. NADP differs from NAD in the 3ʹ-hydroxyl of the adenosine ribose being phosphorylated. Thus, binding of NADP is a priori excluded because of the negatively charged Glu/Asp that interacts with the unmodified ribose hydroxyls in NAD dehydrogenases. Indeed, the replacement of the β2-Asp/Glu is a prerequisite for the switch in specificity to NADP (S11 Fig) [59,60]. Thus, loss of the canonical Glu/Asp underlines the evolution of orthogonal, NADP-dependent dehydrogenases.

The existence of alternative ribose-binding modes with binding energies that are similar to that of the canonical Rossmann mode (Fig 5) and the accordingly wide distribution of binding modes of the noncanonical interactions (as reflected by the interaction angle α; Fig 2B) also support the hypothesis that the canonical Rossmann motif is the outcome of common ancestry and not of convergent evolution. Many structural features are the outcome of strict biophysical constraints, namely of one geometry being highly favored (a deep-well potential energy surface). The negative constraints (steric clashes, loss of resonance energy, etc.) are most dominant in dictating deep-well potentials. This is, for example, the case with the planarity of amide bonds [55]. In contrast, the multiminima potential energy surface for the carboxylate-ribose interaction indicates strong constraints acting only at the edges (around 0° and 180°; Fig 5). This suggests that the conservation of the interaction angle in Rossmann enzymes relates to their divergence from a common ancestor in which this angle was dictated by various factors, including but not limited to the favorable ribose-carboxylate interaction.

### The Ribose-Binding Rossmann Ancestor

**Fig 6:**
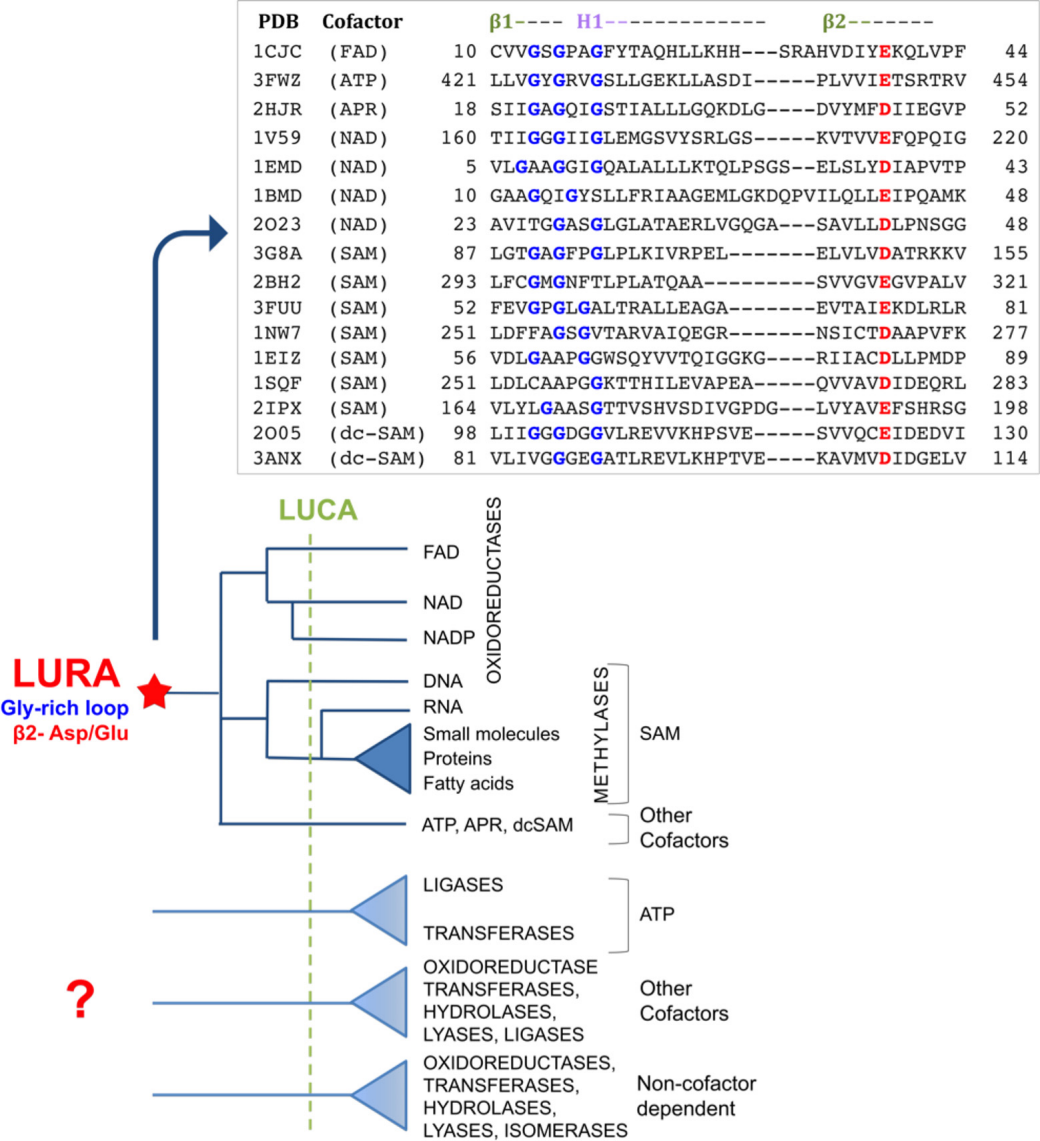
Manual alignment of the β1-H1-β2 segments of representative Rossmann-fold enzymes that possess the canonical motif and a schematic evolutionary tree of the Rossmann fold. Shown are representatives from the four major classes that seem to have diverged from a common ancestor carrying the β2-Asp/Glu motif (Fig 1). The ribose-binding β2-Asp/Glu is in red. As previously noted [5,33], the motif GxGxxG (in blue) is present in almost all the NAD/FAD enzymes, as well as in enzymes utilizing other phosphate-containing cofactors (ATP, AMP, and adenosine-5-diphosphoribose [APR]). In SAM (or dc-SAM) utilizing enzymes, the Gly-rich motif is blurred as expected for a cofactor that does not contain a phosphate group. The schematic tree originates from a presumed last universal Rossmann ancestor (LURA), and it is based on Enzyme Commission (EC) numbers and CATH classification of LUCA’s enzymes (S1 Table). The star designates the presumed common Rossmann ancestor that includes the ribose-(Asp/Glu-β2) and the Gly-rich motifs.

**Fig 7:**
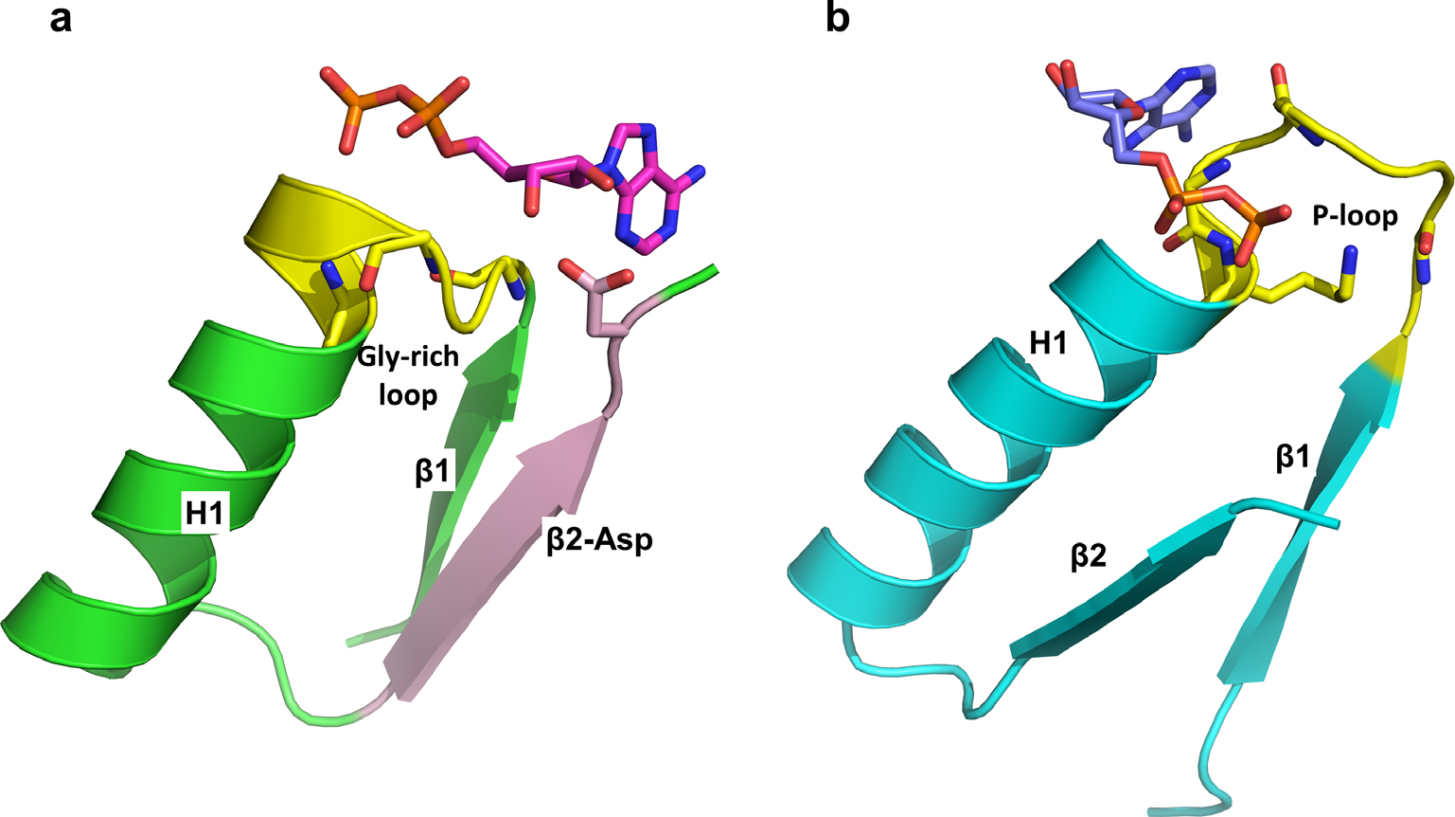
Putative minimal elements underlying the emergence of cofactor-utilizing enzymes. (A) The β1-α1–β2 segment that contains Gly-rich as well as the canonical β2-Asp/Glu interaction comprises the keystone of Rossmann enzymes [40,62] (taken from an NAD-dependent dehydrogenase, PDB 1LDN; see also S13 Fig). Indicated are the Gly-loop (glycines 27, 29, and 32, in yellow) and the canonical ribose ligating Asp at the tip of β2. (B) The P-loop comprises the keystone of the P-loop containing nucleotide triphosphate hydrolases. Shown is the β1-α1–β2 segment taken from a kinase (PDB 2AXN; in complex with ADP). The P-loop (yellow) stems from the first β-strand (β1) and into the first helix (H1). Note that the adenosine di-phosphate moiety, which is common to both cofactors, is bound in opposite directions (the β1-α1–β2 segments were aligned in the same direction).

Common ancestry is the hallmark of Darwinian evolution. Our data support the notion of a primordial Rossmann ancestor in which binding of an adenosine-based cofactor was mediated by the ribose-β2-Asp/Glu interaction, alongside the Gly-loop that resides at the tip of the first strand (β1) (Fig 6, S13 Fig) [24,30,36,39]. The Gly-rich motif binds the phosphate groups of NAD/FAD/adenosine-5ʹ-triphosphate (ATP) (typically, GxGxxG) [5,61]. This motif is also recognizable in methyltransferases, although with low sequence identity because, unlike NAD- and FAD-dependent enzymes, their cofactor, SAM, does not contain a phosphate group (Fig 6). The minimal postulated ancestor therefore spans the Rossmann fold's first two strands and the connecting helix (β1-H1-β2) and includes the Gly-rich and ribose-β2-Asp/Glu interaction (Fig 7A) [40,62]. Our analysis supports a postulated pre-LUCA ancestor that underlined the divergence of at least three major enzyme classes: methyltransferases, NAD(P) and FAD oxireductases [29], and the many superfamilies belonging to these two classes, as well as the divergence of other enzyme families using other adenosine-based cofactors such as ATP (Fig 6). The Gly-rich loop and the ribose-β2-Asp/Glu motif was the keystone of this primordial ancestor [40,62]. Such keystone elements may relate to earlier precursors, possibly shorter polypeptides that contained these binding motifs [5,40,41,43,45] and from which the Rossmann ancestor evolved via a series of duplications, recombination, and fusions [63,64].

#### Cofactor binding—The keystone

The notion of a cofactor binding as the keystone underlying the emergence of the early proteins [5,44,45] is also supported by another ancient fold with a related topology to the Rossmann fold: the P-loop NTPases. Notable in the P-loop NTPases is the exchange between the second and third strands (β2, β3 strand; Fig 1) [5,65,66]. Indeed, the ribose-β2-Asp/Glu interaction is completely absent in this superfamily/fold (Table 1). Instead, this superfamily is underlined by the P-loop, an omnipresent, ancient phosphate-binding motif that appears in many other superfamilies with different folds [5,66–68]. Like Rossmann enzymes, P-loop NTPases make use of ribose-containing cofactors. However, in these enzymes the P-loop comprises the keystone. Not only is the ribose-β2-Asp/Glu missing in P-loop NTPases, but the nucleoside binding orientation is the opposite of the one observed in the Rossmann fold. Curiously, P-loop NTPases have a second conserved motif, the so-called Walker B motif that often comprises an acidic residue following a stretch of hydrophobic ones [69,70]. The latter form a β-strand, as is the case with the Rossmann β2-Asp/Glu motif. However, the Walker B motif is far less conserved than the Rossmann β2-Asp/Glu motif and typically comprises the third strand of the P-loop NTPase fold. Consequently, in P-loop NTPases, the ribose 2ʹ and 3ʹ hydroxyls typically face the solvent rather than interact with protein residues (Fig 7B). Further, the glycine-rich phosphate-binding motifs of these two rudimentary folds comprise mirror images of one another—GxxGxG in P-loop NTPases versus GxGxxG in NAD-dependent Rossmann fold (Fig 7). Thus, despite >3.7 billion y of evolution, these keystones comprise detectable fingerprints of divergent evolution from pre-LUCA ancestors and of the early emergence and evolution of cofactor-utilizing enzymes.

## Methods

### Dataset Assembly

For the study of the individual enzyme classes, all structures belonging to SAM-dependent methyltransferases (SCOP category c.66.1), NAD(P)-binding Rossmann-fold domains (c.2.1), and FAD/NAD-linked oxidoreductases (c.3.1.5) were downloaded from SCOP (v.1.75). Redundant structures of the same protein in which the PDB code was the same for the first three letters/digits and the Glu/Asp residue number was identical were removed. Structures with <2.5 Å resolution were further considered, resulting in 55 methyltransferase (c.66.1) and 315 oxidoreductase (c.2.1 and c.3.15) enzyme domains that were assigned as Rossmann by SCOP (a flowchart describing this analysis is available as S3 Fig). For the systematic analysis of all ribose-binding proteins, we first identified 66 ribose-containing ligands (S2 Table) for which ≥10 nonredundant structures are available in the PDB. We excluded ligands that are part of polynucleotides such as RNA or DNA. All PDB structures that have ribose-containing ligands and <2.5 Å resolution were downloaded, and 80% sequence redundancy was removed with cd-hit [71]. The final dataset comprised 2,949 structures (Table 1) comprising 210 P-loop NTPase structures, 2,313 structures containing ligands with one ribose ring, and 426 structures with ligands such as NAD or FAD that contain two riboses (a flowchart describing this analysis is available as S5 Fig). The four structures with NAD ligands and two bidentate interactions were analyzed separately.

### Geometry and Topology of Ribose Binding

We calculated the distances, angles, and dihedral angles of atoms of interest using the PDB coordinates and custom Perl-scripts. For all retrieved PDB structures, the first chain in the asymmetric unit containing the cofactor was extracted. A random sample indicated that the variability in the distances and angles between different molecules in the asymmetric unit is low, and hence, an arbitrary choice of the first chain containing the cofactor is representative (S1 Text; average standard deviation for the distance is 0.074 Å, and for α is 2.2°). First, all residues that bind the ribose ligands were determined using CSU, and based on whether there is an Asp/Glu residue in the vicinity of the 2’, and 3’-OH of the ribose (≤4 Å). Then, we further characterized the ribose-Asp/Glu interaction and defined four binding modes: canonical bidentate, noncanonical bidentate, monodentate, or “no Asp/Glu interaction.”

The canonical bidentate interaction was defined by four criteria:

1. A bidentate interaction indicated by the distances between both oxygens of the interacting carboxyl moiety and the O2 and O3 of the ribose is ≤3.4 Å.
2. The plane angle (α; calculated as described in S1 Text) is in the range of 90° to 140°.
3. The interacting Asp/Glu residue is located at the tip of a β-strand. To identify the latter, secondary structure was assigned by dssp (H: alpha helix, E: strand, T: turn, S: bend, L: loop, G: 3/10-helix); the location criterion was defined as met when the interacting Asp/Glu comprised the last position within a strand or the next consecutive residue after a strand. For the initial analysis of individual families (c.66.1, c.2.1 and c.3.1.5), a more stringent threshold was set up for the first criterion whereby at least one of the distances between the hydroxyl 2ʹ-and 3ʹ-oxygens of the ribose was ≤3 Å.
4. The ribose’s furanose ring conformation is in an envelope form, mainly the E_1_ and ^2^E conformations.

Noncanonical bidentate interaction was assigned to structures meeting criterion (i), namely structures with a bidentate interaction yet with the plane angle being <90° or >140° and the interacting Asp/Glu not located at the tip of a β-strand.

Monodentate interactions were assigned to structures with a single putative H-bond interaction between an Asp/Glu carboxylate and either the 2ʹ or the 3ʹ-hydroxyl groups. A more generous cutoff distance of ≤4 Å was taken here than for the bidentate interactions (≤3.4 Å) because the latter, and especially the canonical bidentate interactions, tend to be much tighter (average distance = 2.7 Å; S2B Fig). Finally, no Glu/Asp interaction was ascribed to structures where no carboxylate was found within 4 Å of either the 2ʹ or the 3ʹ-hydroxyl groups of the bound ribose.

### Fold Annotation

When available, we retrieved the CATH and SCOP classification for the PDB structures in our dataset. Assignments of Rossmann fold were derived from CATH topology 3.40.50 (CATH_v3.5.0, version date: 20.09.2013, was used for this analysis). However, as explained in the main text, we separately analyzed superfamily 3.40.50.300, the P-loop containing nucleotide triphosphate hydrolases that are usually not considered as Rossmann. For SCOP, categories c.66.1, c.2.1, c.3.1, and c.4.1 were assigned as Rossmann. Including both CATH and SCOP databases significantly increased the fraction of structures with annotated fold (e.g., for structures containing one ribose ligands, the CATH database assigns 207 proteins as Rossmann, and addition of SCOP added another 85). About 46% of structures had neither a CATH nor a SCOP annotation (1,354/2,949). We therefore manually inspected a randomly chosen subset of the structures that possess the canonical interaction. We confirmed these as belonging to the Rossmann fold by identifying the canonical 3-2-1-4-5-6 topology of β-strands, or as Rossmann-like by identifying structures in which the last β strand (β6) is missing (S5 Table).

### Role of Glu29 in Methyltransferase M.*Hae*III

A variant of M.*Hae*III containing four stabilizing mutations and with wild-type-like activity was the starting point for generating the Glu29 mutants [72]. The pASK-IBA3+vector (IBA, ampicillin resistance) plasmid containing the gene for the stabilized M.*Hae*III was used as a template for PCR amplification. Mutants in position 29 were constructed by site-directed mutagenesis. The Glu codon was replaced with the Gln codon (CAA), Thr codon (ACC), Leu codon (CTG), Asp codon (GAT), Trp codon (TGG), Ala codon (GCG), Val codon (GTG), or Ser codon (AGC). The mutant encoding plasmids were transformed into *E*. *coli* MC1061, [mcrA0 relA1mcrB1 hsdR2 (r-m+; in which DNA methylation is not toxic) bearing the GroEL/ES encoding plasmid pGro7 (chloramphenicol resistance; Takara) to assist the folding of compromised mutants [72]. Transformants were selected by growth in the presence of ampicillin and chloramphenicol. The methyltransferase activity was tested by treatment of the extracted plasmid with the cognate restriction enzyme, HaeIII. The level of plasmid protection by virtue of methylation by M.*Hae*III was determined by gel analysis. Bacteria were grown with no inducer or under induction (0.2 μg/ml anhydrotetracycline) and with 0.05% arabinose for induction of GroEL/ES expression. Wild-type M.*Hae*III gave full protection even when basally expressed (no inducer). Time-dependent in vitro methylation assays were performed with purified enzyme variants (0.1–8 μM) essentially as described [73], using H^3^-labeled SAM (0.1–8 μM) and DNA substrate carrying nine methylation GGCC sites per molecule at 2.5 nM.

### QM Calculations

We carried out quantum mechanical electronic structure calculations on models M1 and M2 (S1 Text) by using the M06-2X/6-31+G(d,p) [74,75] model chemistry including the effect of aqueous solvent by using the SMD solvation model [76]. All electronic structure calculations were performed with *Gaussian09* [77]. We performed an exhaustive conformational search for model M1 (Fig 4A). Starting from the lowest-energy optimized structures obtained with model M1, namely ^2^E-endo and ^3^E-exo, we carried out a relaxed potential energy surface scan along the coordinate defined by α (see Fig 5A). In the scan, all degrees of freedom were optimized with the exception of the angle α. This was accomplished by interfacing the *Gaussian 09* program^49^ with a utility program we wrote that allows a constraint on the angle between two vectors. For model M2 (Fig 5B), after carrying out a conformational analysis of the molecule of adenosine and an analysis to find the best conformations that lead to a double hydrogen bond with a molecule of acetate, three fully optimized structures of model M2, denoted as g-t, g-a, and t-t, were found. These structures were taken as initial geometries to explore the potential energy surface (PES). The PES was explored by a combination of successive relaxed energy minimization scans along two angles and a dihedral angle that equals to perform a scan along the angle α (see S1 Text).

## Supporting Information

S1 Data Numerical data underlying Fig 2B. (XLSX)

S2 Data Numerical data underlying Fig 4C. (XLSX)

S3 Data Numerical data underlying Fig 5. (XLSX)

S4 Data Numerical data underlying S2 Fig. (XLSX)

S1 Fig PDB structures of different methyltransferases presenting the canonical motif. SAM or SAH cofactors are shown in green sticks, β2 strand of Rossmann fold in orange, and the interacting Asp/Glu with the hydroxyls’ ribose in orange sticks. (A) The catalytic domain of bacterial DNA methylase M.*Hha*I (PDB 1SKM); (B) human DNA methylase Dnmt3a (PDB 2QRV); (C) human DNA methylase Dnmt1 (PDB 3AV6). (D) An mRNA methylase (PDB 1RI4). (E) An N5-glutamine methylase (PDB 1NV8). (F) Catechol methylase (PDB 3BWM).
(TIF)

S2 Fig Distribution of the geometrical parameters of the canonical interaction for SCOP families c.66.1 (SAM) and c.2.1 (NAD). (A) Definition of the geometrical parameters. (B) Distribution of the bond lengths between the hydroxyl group of the ribose and the closest oxygen of the carboxylate of the Glu/Asp. (C) Distribution between the angle of the hydroxyl bond of the ribose and the carboxylate. (D) Distribution of the dihedrals of O-C1-C2-C3 and C1-C2-C3-C4. The distribution highlights a distorted envelope 2ʹ endo conformation of the ribose for most of the structures. Data for all PDB entries are provided in S4 Data.
(PNG)

S3 Fig Flowchart for the initial identification and analysis of the SCOP families c.66.1 (SAM-dependent enzymes), c.2.1 (NAD-dependent enzymes), and c.3.1.5 (FAD-dependent enzymes). (PNG)

S4 Fig The structure of catechol methyltransferase (PDB 3BWM). The 2β strand is in orange, the E90 interacting with the hydroxyls of the ribose is shown as an orange stick, and Mg^2+^ is shown in the green sphere. The electron density map of the ribose is highlighted in blue, showing the ^2^E-endo conformation of the ring.
(PNG)

S5 Fig Flowchart describing the systematic analysis of all ribose-bound proteins in the PDB. (PNG)

S6 Fig Representative examples of noncanonical PDBs structures having a bidentate E/D interaction. PDBs, corresponding cofactor and α angle: (A) 1HO5, ADN (adenosine) (29°); (B) 2J9L, ATP (adenosine-5ʹ-triphosphate) (27°); (C) 3S2U, UD1 (uridine-diphosphate-N-Acetylglucosamine) (19°); (D) 1K9Y, AMP (adenosine monophosphate) (127°); (E) 2ATV, GDP (guanosine-5ʹ-diphosphate) (27°); example of P-loop containing nucleoside triphosphate hydrolases); (F) 1SIW, GDP (137°); (G) 3TE5, NAI (1,4-dihydronicotinamide adenine dinucleotide) (18°); (H) 1I7L, ATP (43°); (I) 4B45 GSP (5ʹ-guanosine-diphosphate-monothiophosphate) (29°).
(PNG)

S7 Fig Two examples of structures for which the Rossmann fold has been manually assigned. The basic Rossmann fold is altered primarily by addition of other elements. The β strands belonging to Rossmann are colored differently than the main structure: β1 in blue, β2 in green, β3 in magenta, β4 in orange, and β5 in wheat. The cofactors and the interacting E/D are in sticks. (A) PDB 3UCL binding FAD. The zoom-in view depicts the ribose’s cofactor binding site. (B) PDB 3CGD is constituted by two Rossmann subunits binding NAD and FAD. The zoom-in views depict the two subunits binding the ribose of the corresponding cofactors.
(PNG)

S8 Fig Canonical and noncanonical interactions coinciding in the same Rossmann enzyme. Cartoon structure of L-2-hydroxyisocaproate dehydrogenase (PDB 1HYH). The cofactors are shown in the green sticks; the interacting D45 (canonical) and E110 (noncanonical) are shown as sticks.
(PNG)

S9 Fig Crystal structure of 4-((1R,2S,4R,5S)-(1,2,5-Trihydroxy-3-oxabicyclo(3.3.0)octane)-4-spiro-1ʹ-(2ʹ-oxocyclopentane)-2-yl)butanoic acid (CDB JOWZUQ). Highlighted are the distances between the vicinal diol and the oxygens of the carboxylic acid.
(PNG)

S10 Fig Human phosphoglyceraldehyde kinase (PDB 2X13). The backbone is shown in the cartoon, the cofactor and residue E344 in sticks, and the Mg^2+^ in the green sphere. The loop that follows the fourth β-strand (4β) and that carries the bidentate interacting Glu344, as well as the 4β, are shown in pink.
(PNG)

S11 Fig Zoom-in view of the homologous SAM and NADP Rossmann enzymes noted by Xie and Bourne [19]. Although these Rossmann-fold enzymes bind different adenosine-containing ligands, Xie and Bourne noted that the adenosine moieties are well aligned, suggesting that these families share a common ancestry. (A) SAM-dependent methyl transferases (PDB 1ZQ9). (B) Carbonyl reductase (PDB 1CYD). (C) Overlapping of the two structures highlights the interaction of E85 (green stick) on 2β tip interacting with SAM’s ribose with the canonical motif and R39 (cyan sticks) on 2β tip interacting with the phosphate moiety of NADP.
(PNG)

S12 Fig A Rossmann-fold dehydrogenase (PDB 3KV3). Asp34 that stems from the second β-strand (2β) mediates the bidentate interaction with the ribose’s 2ʹ and 3ʹ-hydroxyls.
(PNG)

S13 Fig A representative manual alignment of the β1-H1-β2 region of various Rossmann-fold enzymes with the canonical ribose-β2 (Asp/Glu) motif. Among the structures with the canonical motif, a sample of 50 PDB structures was randomly selected. Following standard alignment, with Multiple Sequence Comparison by Log-Expectation (MUSCLE), sequences were grouped by the cofactor. The phosphate-containing cofactors depicted are as follows: ATP, adenosine-5ʹ-triphosphate; AMP, adenosine monophosphate, and APR, adenosine-5-diphosphoribose; FAD, flavin adenine dinucleotide; and NAD, nicotinamide adenine dinucleotide; the non-phosphate-containing cofactors are as follows: SAM, S-adenosylmethionine, and dc-SAM, Adenosylmethioninamine.
(PNG)

S1 Table LUCA representatives belonging to the Rossmann fold and classified according to the CATH annotation [48] or three-digit EC number [49]. (PNG)

S2 Table Cofactors found in the structures considered in this study. (TIF)

S3 Table Details of manually analyzed structures of representative structures from Table 1. (PNG)

S4 Table List of the Rossmann-fold structures in S2B Fig with α angle between 0° and 60° (*n* = 66/811). The interacting E/D is located on different secondary structure elements and never at the tip of the β2 strand.
(PNG)

S5 Table Structures with no SCOP and CATH annotations that appear to belong to the Rossmann fold. (PNG)

S6 Table Details of the carboxylate-ribose interaction for the 279 structures with no fold assigned. (PNG)

S7 Table Rossmann-fold enzymes utilizing NAD (cofactors with two riboses). Noted are the distances of the interacting E/D at the top of the β2 strand to the ribose hydroxyls. In all these cases, the interacting ribose belongs to the adenosine moiety of these cofactors and not to the nicotinamide (NAD, or NADH—annotated as NAI) or the free ribose (in APR).
(PNG)

S8 Table M.*Hae*III, glyceraldehyde-3-phosphate dehydrogenase (GAPDH) [51] and sarcosine oxidase (SoxA) [52] kinetic parameters for the wild-type enzyme and mutants of the ribose binding E/D. (PNG)

S9 Table Summary of the criteria for the characterization of the various carboxylate-ribose interactions. (PNG)

S1 Text Quantum mechanical calculations. (DOCX)

## Abbreviations

AMP: adenosine monophosphate
APR: adenosine-5-diphosphoribose
ATP: adenosine-5ʹ-triphosphate
CATH: Class Architecture Topology Homologous superfamilies
EC: Enzyme Commission
ECOD: Evolutionary Classification of Protein Domains
FAD: flavin adenine dinucleotide
GAPDH: glyceraldehyde-3-phosphate dehydrogenase
LUCA: last universal common ancestor
LURA: last universal Rossmann ancestor
NAD: nicotinamide adenine dinucleotide
NADP: nicotinamide adenine dinucleotide phosphate
NTPase: nucleoside triphosphatase
MUSCLE: Multiple Sequence Comparison by Log-Expectation
PDB: Protein Data Bank
PES: potential energy surface
SAM: S-adenosyl methionine
SCOP: Structural Classification of Proteins
SMD: Solvation Model based on Density
