# Supplementary figures and images for: An Ancient Fingerprint Indicates the Common Ancestry of Rossmann-Fold Enzymes Utilizing Different Ribose-Based Cofactors
Source: PLoS Biol. 2016 Mar 3;14(3):e1002396. doi: 10.1371/journal.pbio.1002396 (PMC4777477; doi:10.1371/journal.pbio.1002396)

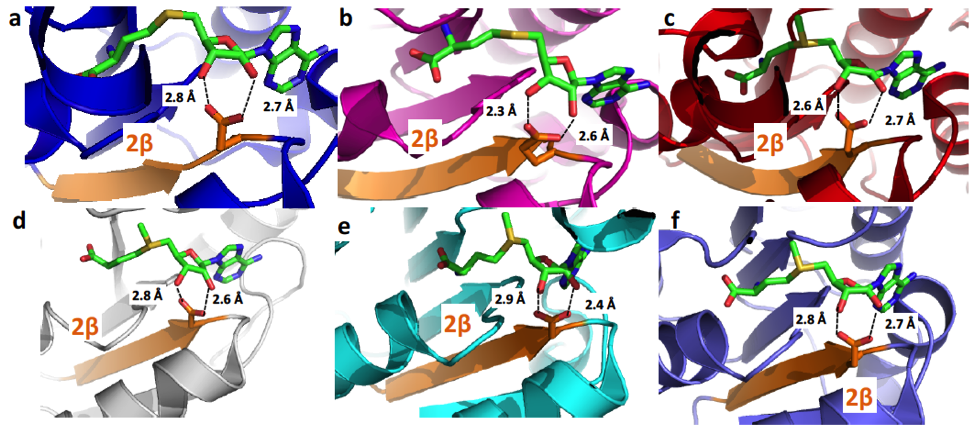

Supplement: S1 Fig — SAM or SAH cofactors are shown in green sticks, β2 strand of Rossmann fold in orange, and the interacting Asp/Glu with the hydroxyls’ ribose in orange sticks. (A) The catalytic domain of bacterial DNA methylase M.HhaI (PDB 1SKM); (B) human DNA methylase Dnmt3a (PDB 2QRV); (C) human DNA methylase Dnmt1 (PDB 3AV6). (D) An mRNA methylase (PDB 1RI4). (E) An N5-glutamine methylase (PDB 1NV8). (F) Catechol methylase (PDB 3BWM). (TIF) [file pbio.1002396.s005.tif]

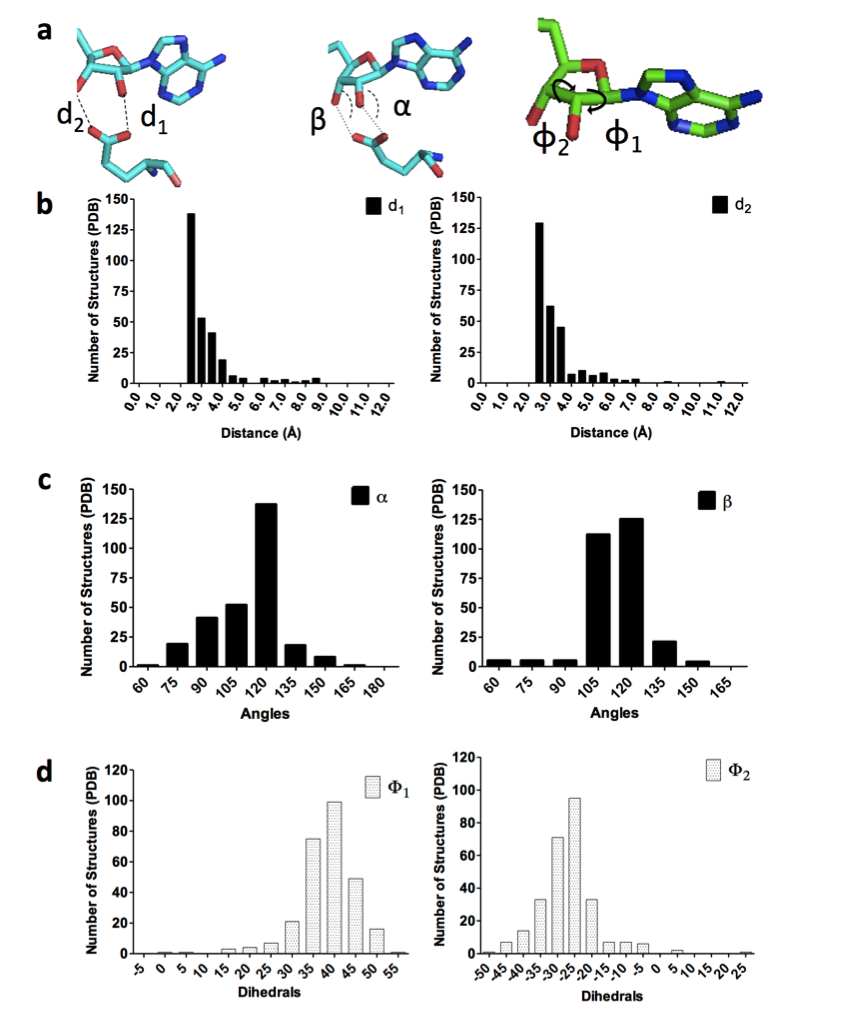

Supplement: S2 Fig — (A) Definition of the geometrical parameters. (B) Distribution of the bond lengths between the hydroxyl group of the ribose and the closest oxygen of the carboxylate of the Glu/Asp. (C) Distribution between the angle of the hydroxyl bond of the ribose and the carboxylate. (D) Distribution of the dihedrals of O-C1-C2-C3 and C1-C2-C3-C4. The distribution highlights a distorted envelope 2ʹ endo conformation of the ribose for most of the structures. Data for all PDB entries are provided in S4 Data. (PNG) [file pbio.1002396.s006.png]

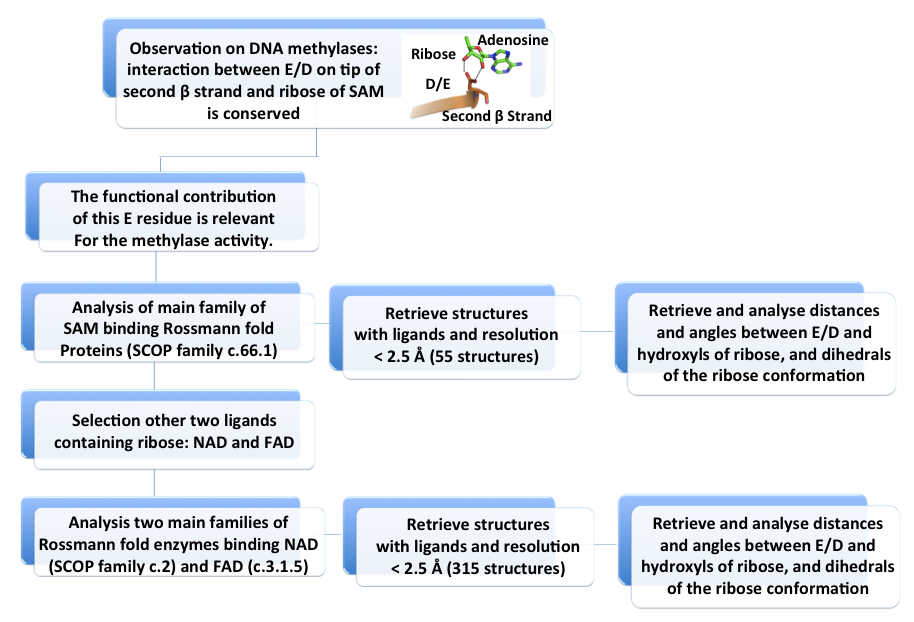

Supplement: S3 Fig — (PNG) [file pbio.1002396.s007.png]

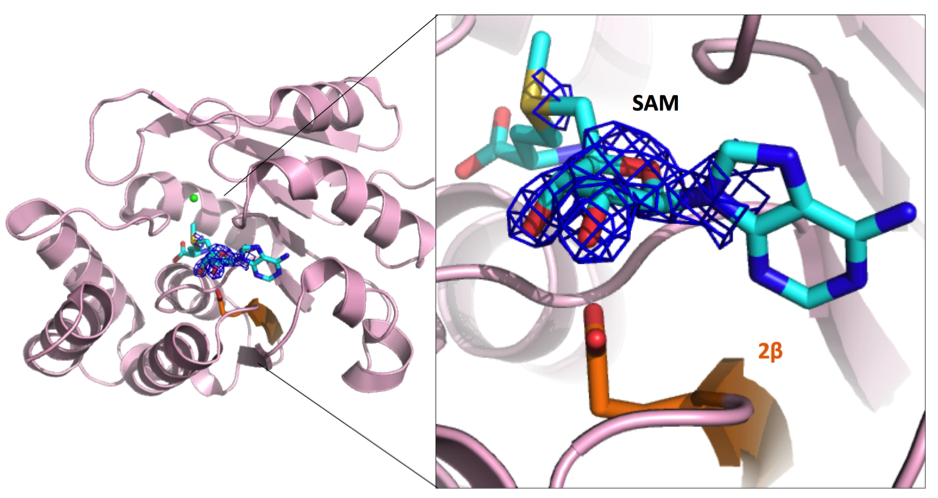

Supplement: S4 Fig — The 2β strand is in orange, the E90 interacting with the hydroxyls of the ribose is shown as an orange stick, and Mg2+ is shown in the green sphere. The electron density map of the ribose is highlighted in blue, showing the 2E-endo conformation of the ring. (PNG) [file pbio.1002396.s008.png]

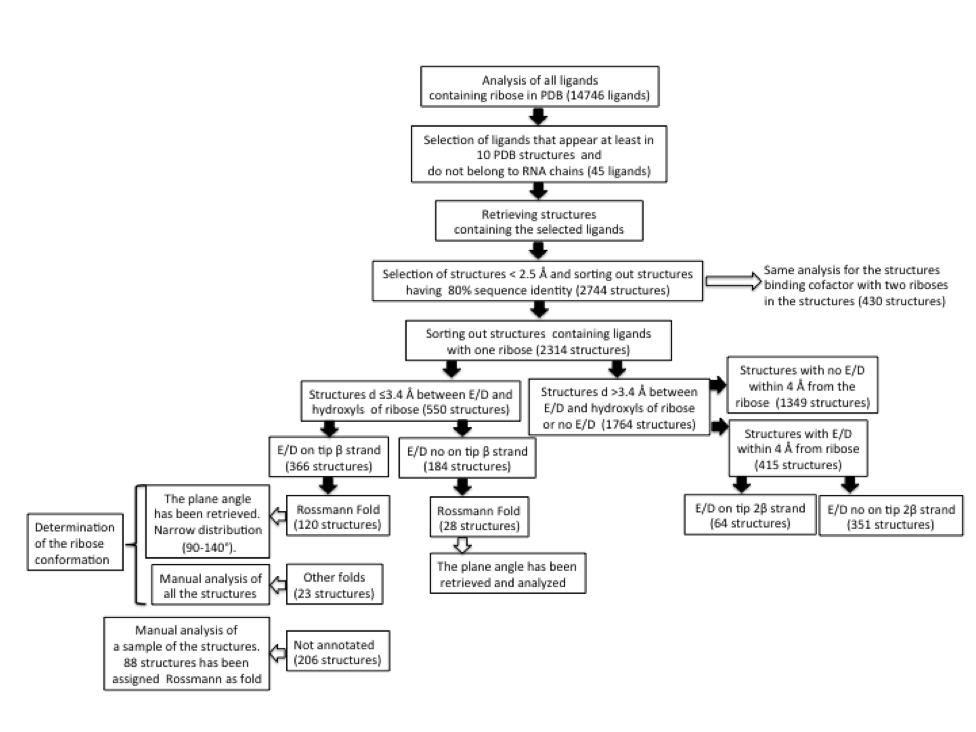

Supplement: S5 Fig — (PNG) [file pbio.1002396.s009.png]

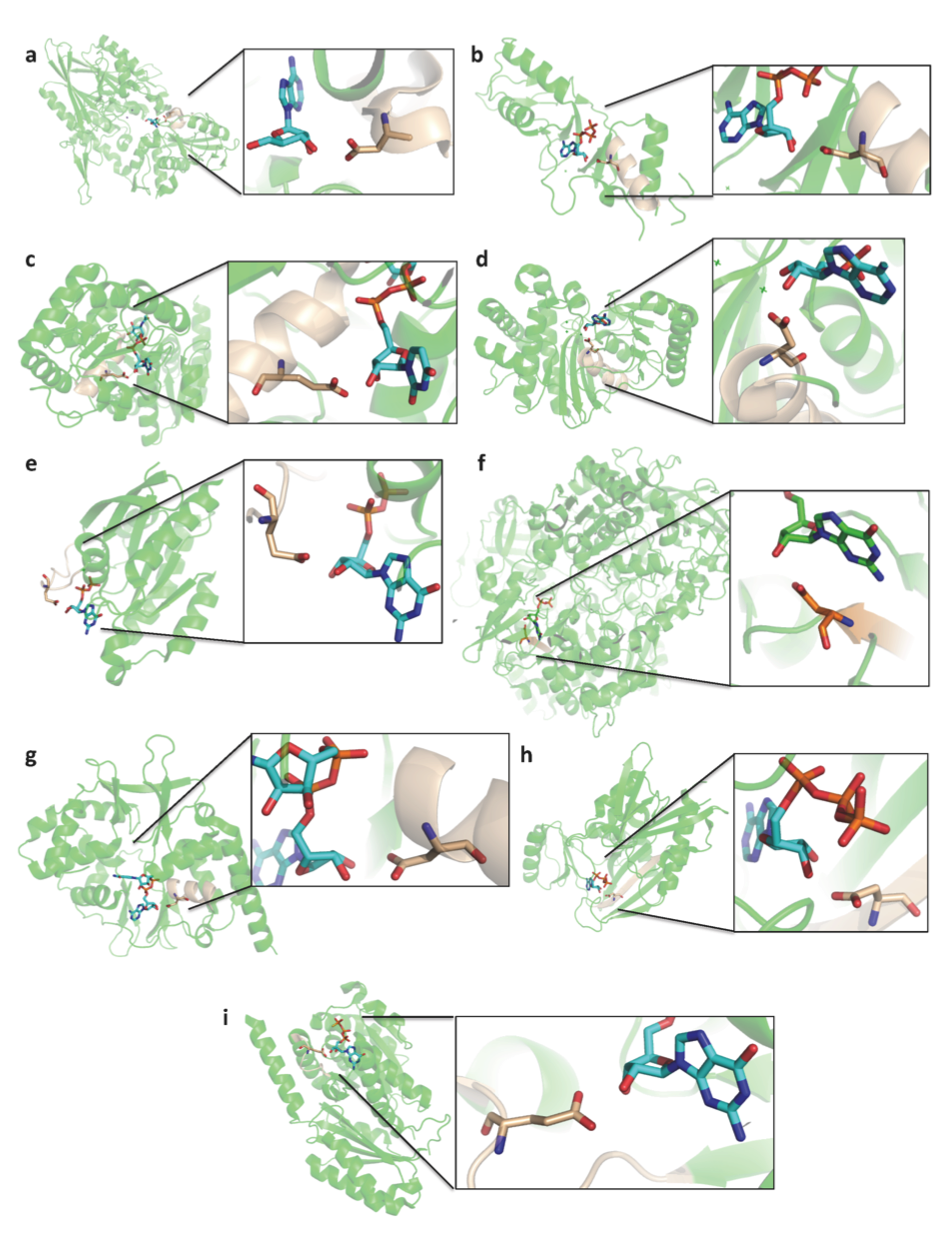

Supplement: S6 Fig — PDBs, corresponding cofactor and α angle: (A) 1HO5, ADN (adenosine) (29°); (B) 2J9L, ATP (adenosine-5ʹ-triphosphate) (27°); (C) 3S2U, UD1 (uridine-diphosphate-N-Acetylglucosamine) (19°); (D) 1K9Y, AMP (adenosine monophosphate) (127°); (E) 2ATV, GDP (guanosine-5ʹ-diphosphate) (27°); example of P-loop containing nucleoside triphosphate hydrolases); (F) 1SIW, GDP (137°); (G) 3TE5, NAI (1,4-dihydronicotinamide adenine dinucleotide) (18°); (H) 1I7L, ATP (43°); (I) 4B45 GSP (5ʹ-guanosine-diphosphate-monothiophosphate) (29°). (PNG) [file pbio.1002396.s010.png]

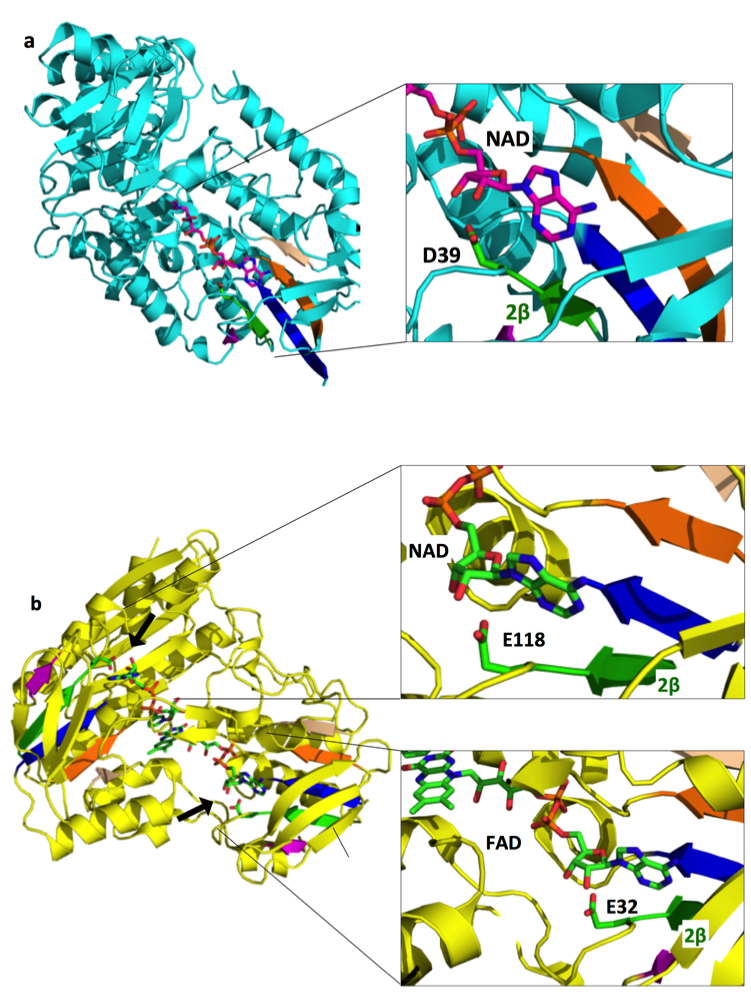

Supplement: S7 Fig — The basic Rossmann fold is altered primarily by addition of other elements. The β strands belonging to Rossmann are colored differently than the main structure: β1 in blue, β2 in green, β3 in magenta, β4 in orange, and β5 in wheat. The cofactors and the interacting E/D are in sticks. (A) PDB 3UCL binding FAD. The zoom-in view depicts the ribose’s cofactor binding site. (B) PDB 3CGD is constituted by two Rossmann subunits binding NAD and FAD. The zoom-in views depict the two subunits binding the ribose of the corresponding cofactors. (PNG) [file pbio.1002396.s011.png]

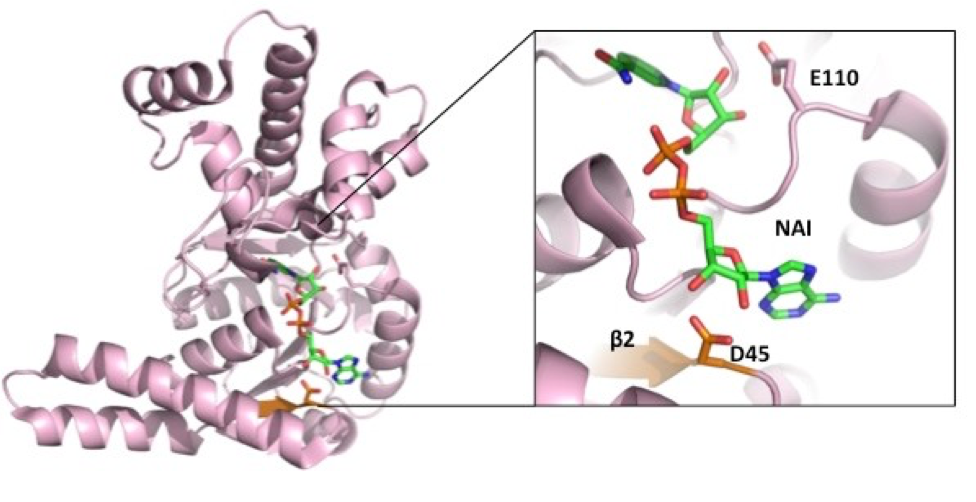

Supplement: S8 Fig — Cartoon structure of L-2-hydroxyisocaproate dehydrogenase (PDB 1HYH). The cofactors are shown in the green sticks; the interacting D45 (canonical) and E110 (noncanonical) are shown as sticks. (PNG) [file pbio.1002396.s012.png]

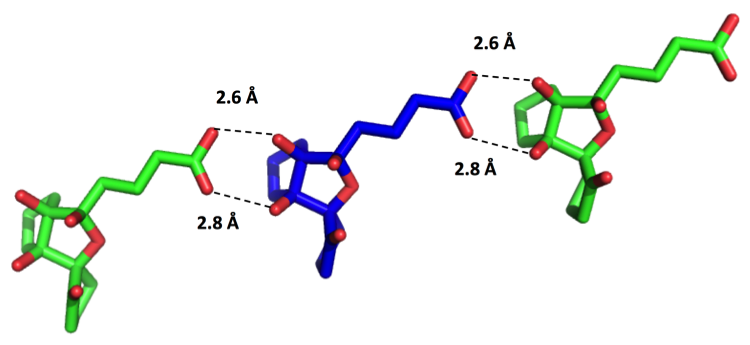

Supplement: S9 Fig — Highlighted are the distances between the vicinal diol and the oxygens of the carboxylic acid. (PNG) [file pbio.1002396.s013.png]

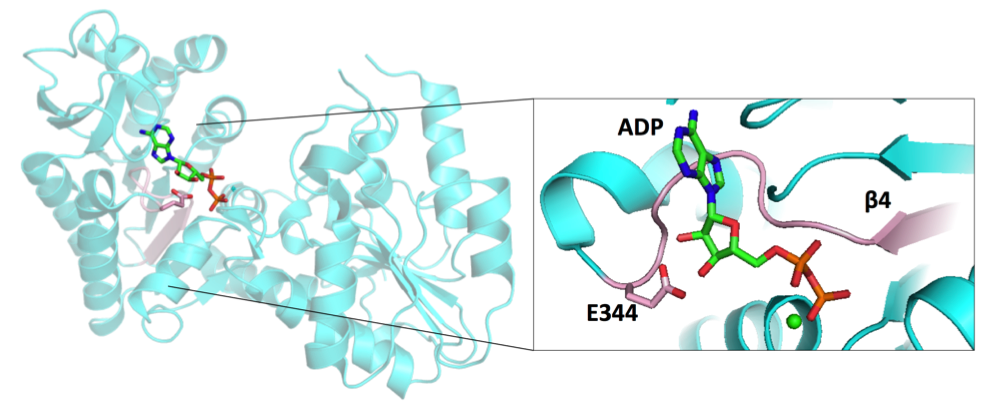

Supplement: S10 Fig — The backbone is shown in the cartoon, the cofactor and residue E344 in sticks, and the Mg2+ in the green sphere. The loop that follows the fourth β-strand (4β) and that carries the bidentate interacting Glu344, as well as the 4β, are shown in pink. (PNG) [file pbio.1002396.s014.png]

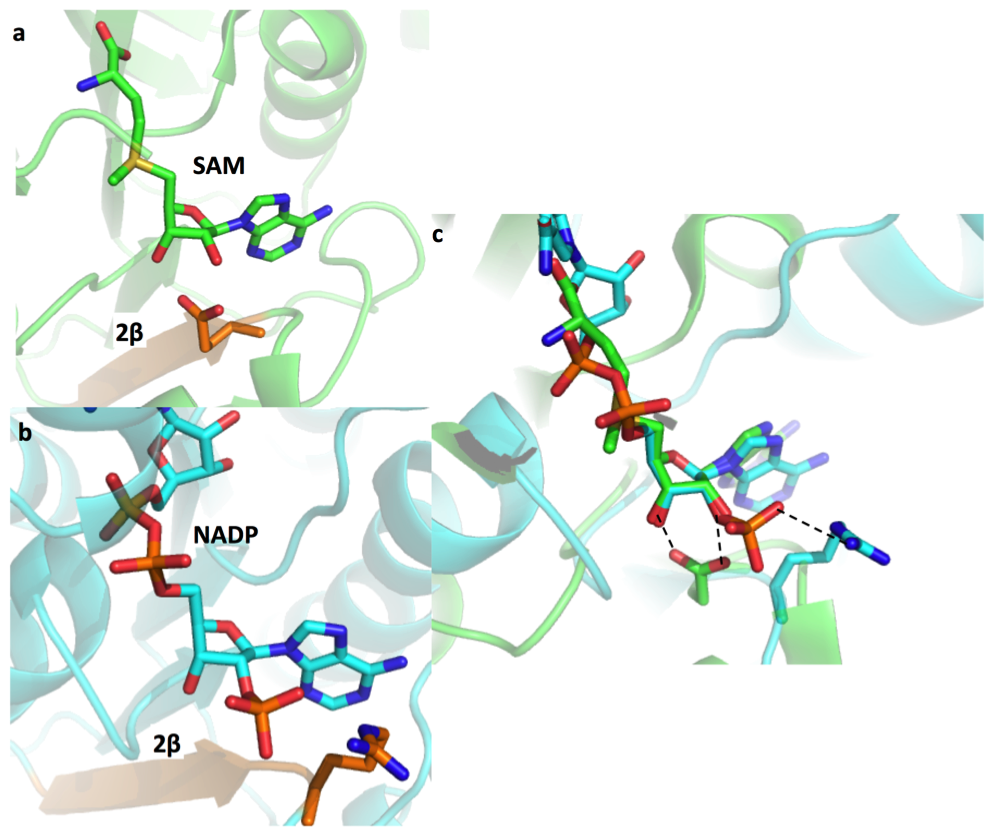

Supplement: S11 Fig — Although these Rossmann-fold enzymes bind different adenosine-containing ligands, Xie and Bourne noted that the adenosine moieties are well aligned, suggesting that these families share a common ancestry. (A) SAM-dependent methyl transferases (PDB 1ZQ9). (B) Carbonyl reductase (PDB 1CYD). (C) Overlapping of the two structures highlights the interaction of E85 (green stick) on 2β tip interacting with SAM’s ribose with the canonical motif and R39 (cyan sticks) on 2β tip interacting with the phosphate moiety of NADP. (PNG) [file pbio.1002396.s015.png]

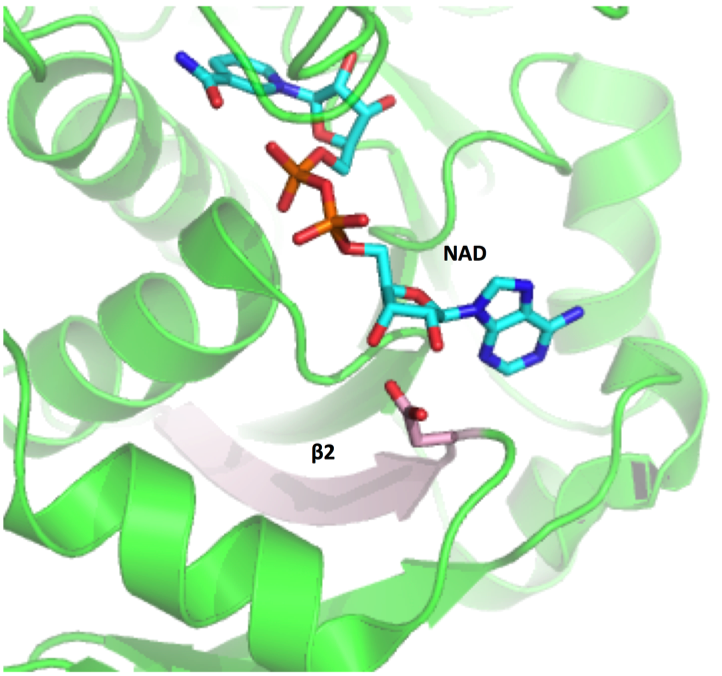

Supplement: S12 Fig — Asp34 that stems from the second β-strand (2β) mediates the bidentate interaction with the ribose’s 2ʹ and 3ʹ-hydroxyls. (PNG) [file pbio.1002396.s016.png]

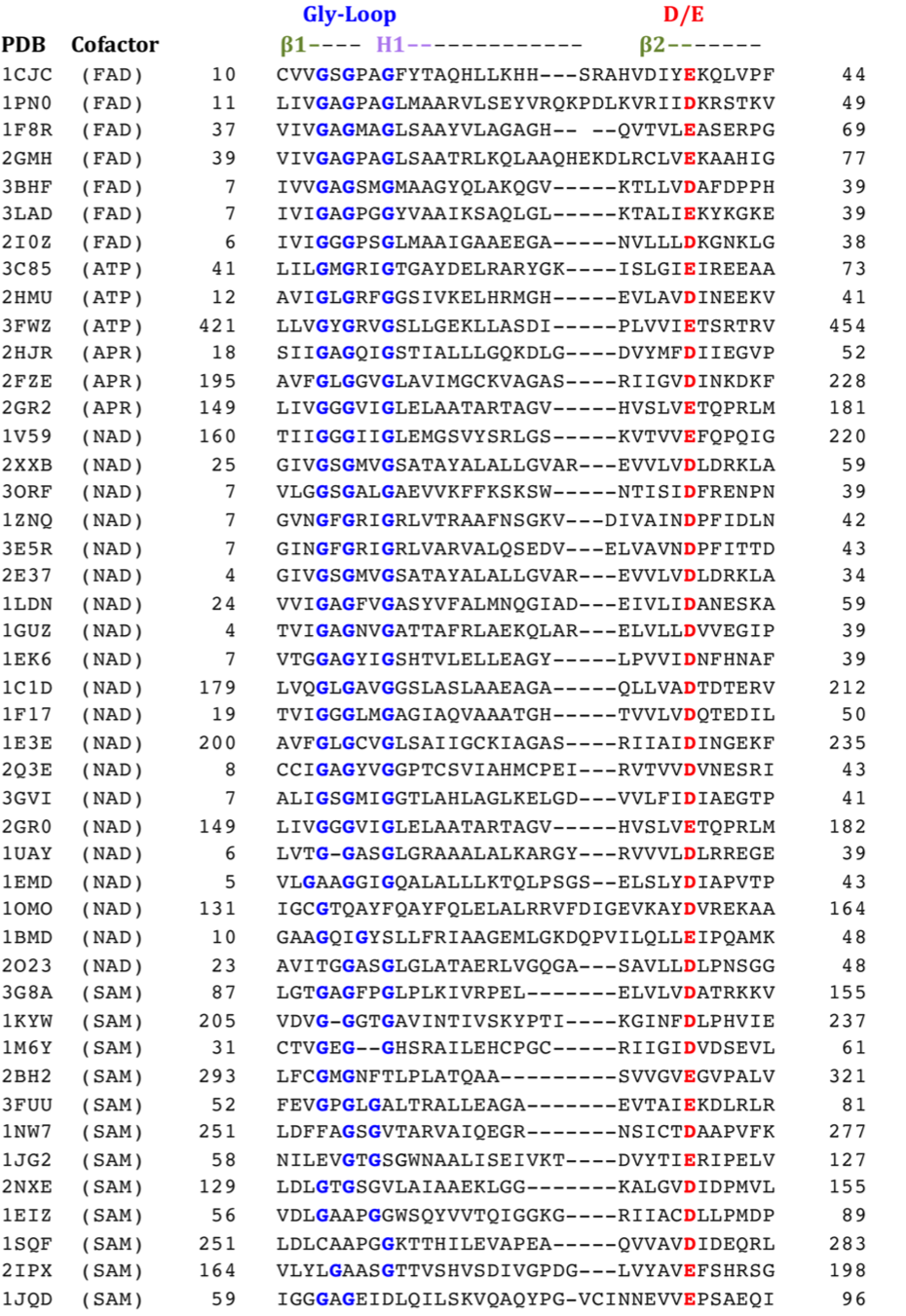

Supplement: S13 Fig — Among the structures with the canonical motif, a sample of 50 PDB structures was randomly selected. Following standard alignment, with Multiple Sequence Comparison by Log-Expectation (MUSCLE), sequences were grouped by the cofactor. The phosphate-containing cofactors depicted are as follows: ATP, adenosine-5ʹ-triphosphate; AMP, adenosine monophosphate, and APR, adenosine-5-diphosphoribose; FAD, flavin adenine dinucleotide; and NAD, nicotinamide adenine dinucleotide; the non-phosphate-containing cofactors are as follows: SAM, S-adenosylmethionine, and dc-SAM, Adenosylmethioninamine. (PNG) [file pbio.1002396.s017.png]

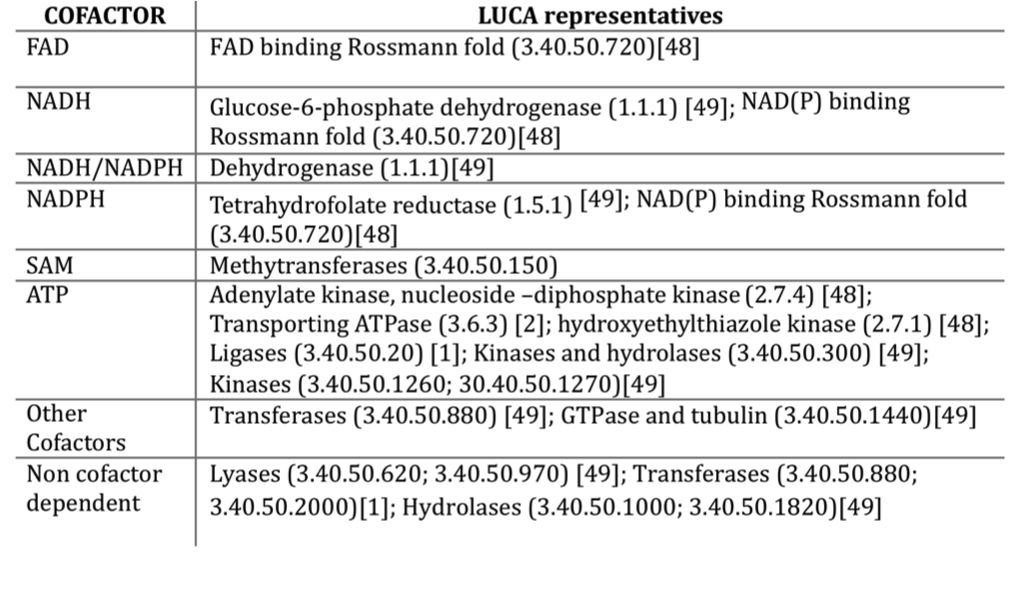

Supplement: S1 Table — (PNG) [file pbio.1002396.s018.png]

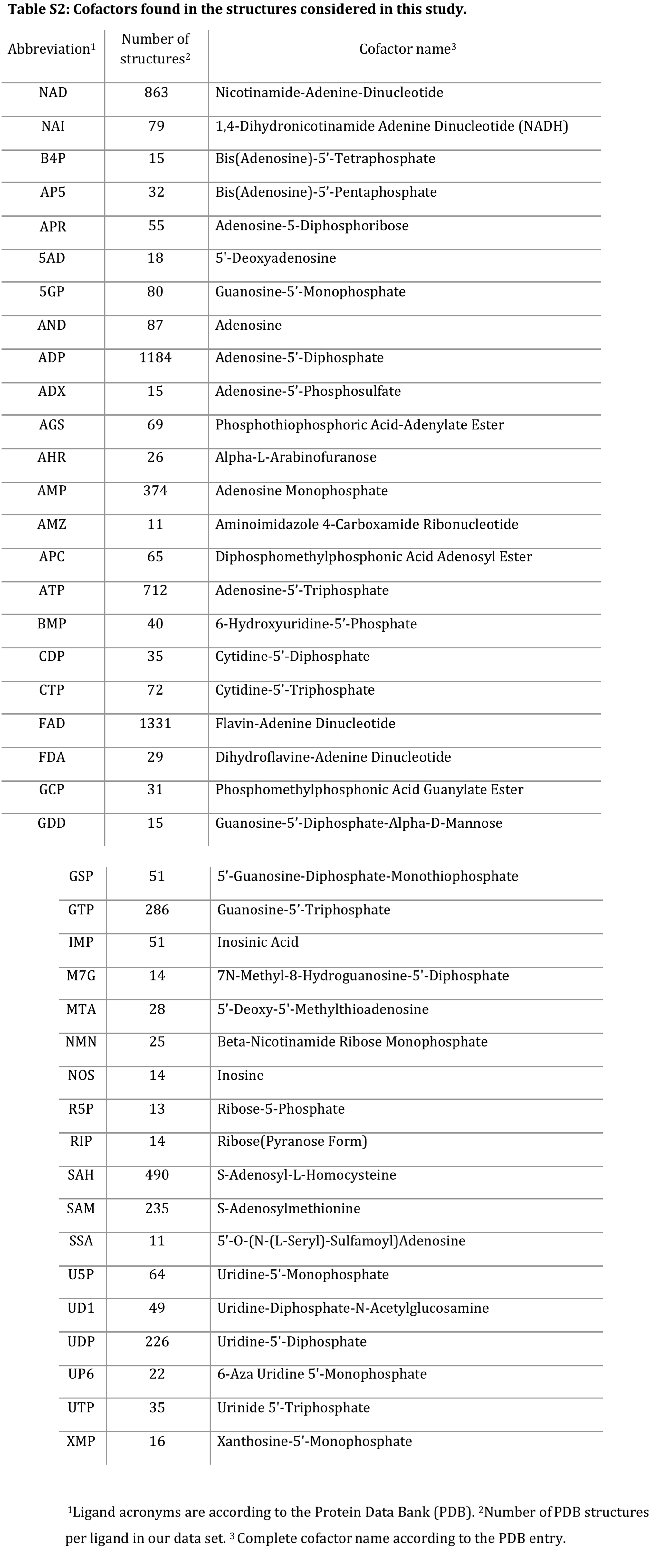

Supplement: S2 Table — (TIF) [file pbio.1002396.s019.tif]

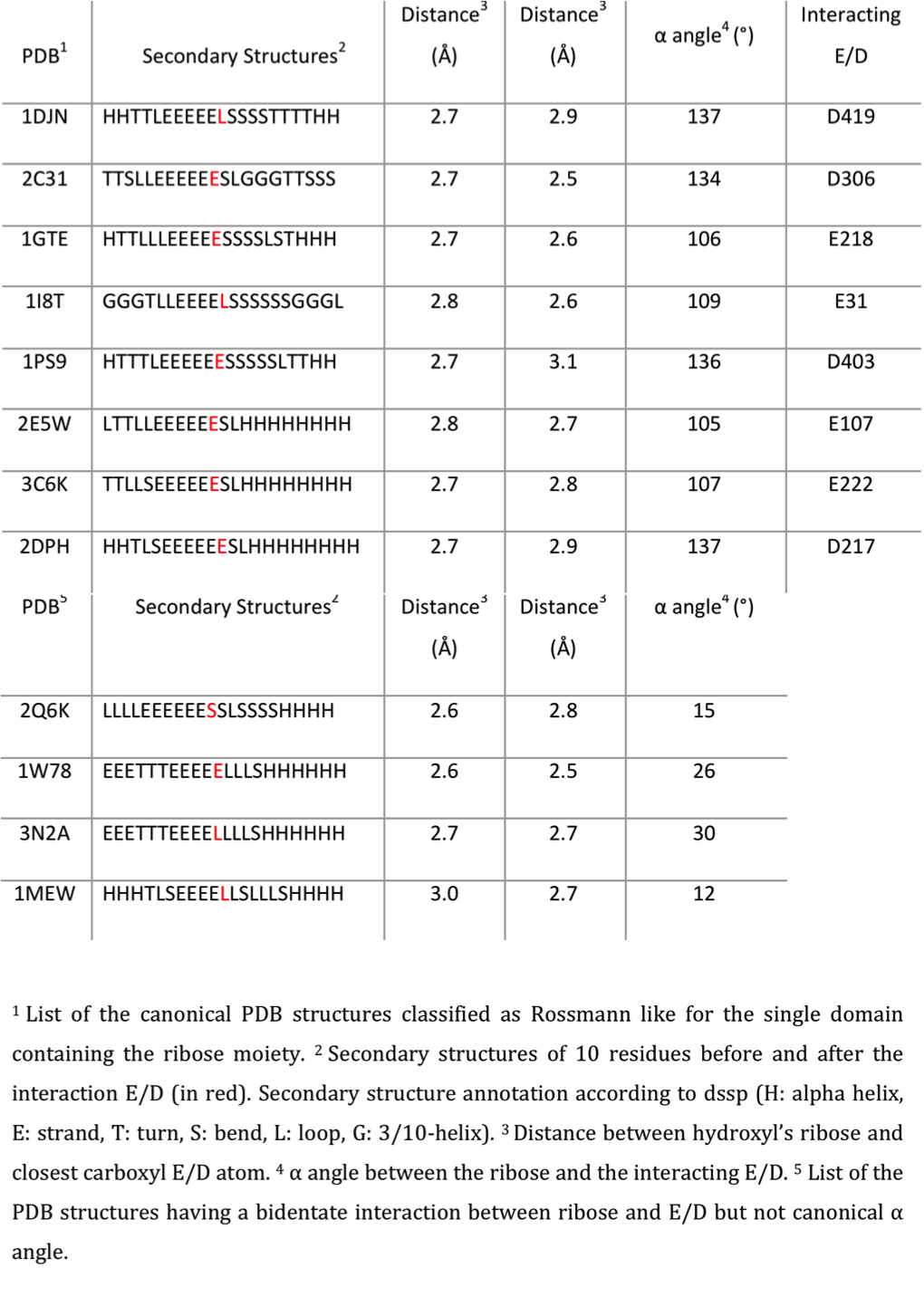

Supplement: S3 Table — (PNG) [file pbio.1002396.s020.png]

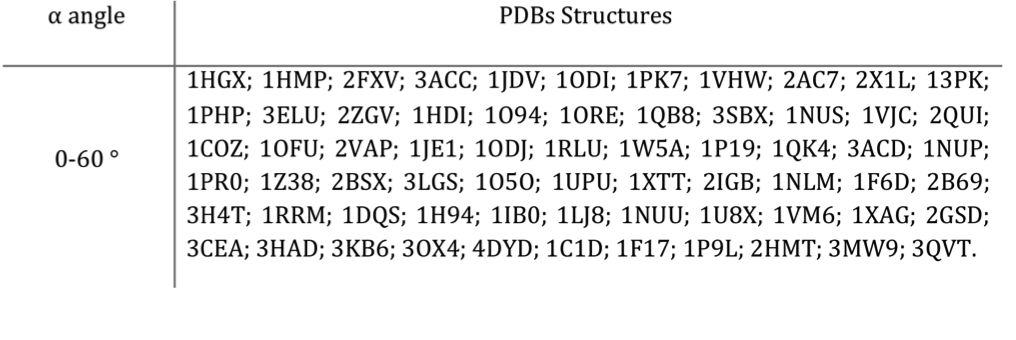

Supplement: S4 Table — The interacting E/D is located on different secondary structure elements and never at the tip of the β2 strand. (PNG) [file pbio.1002396.s021.png]

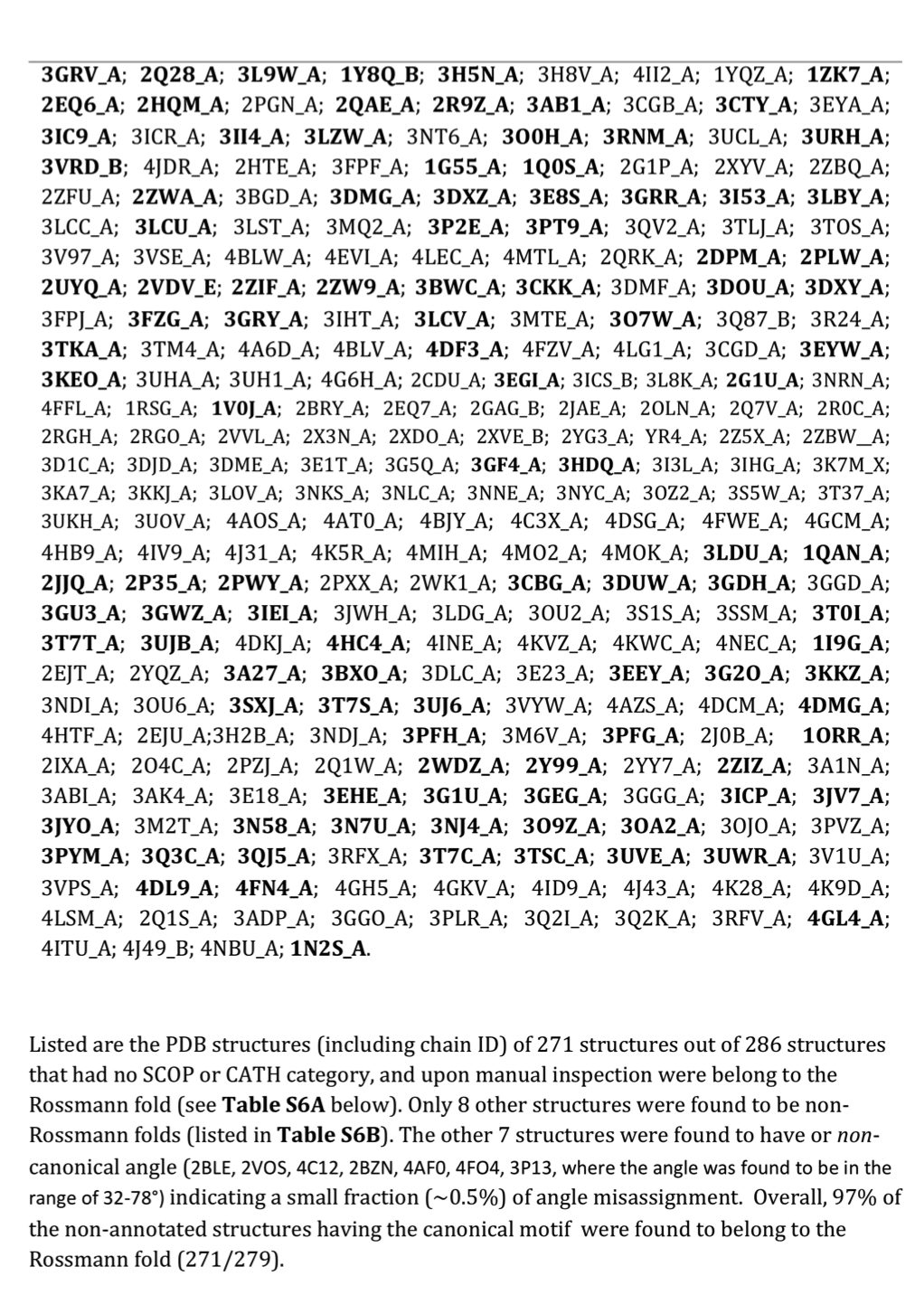

Supplement: S5 Table — (PNG) [file pbio.1002396.s022.png]

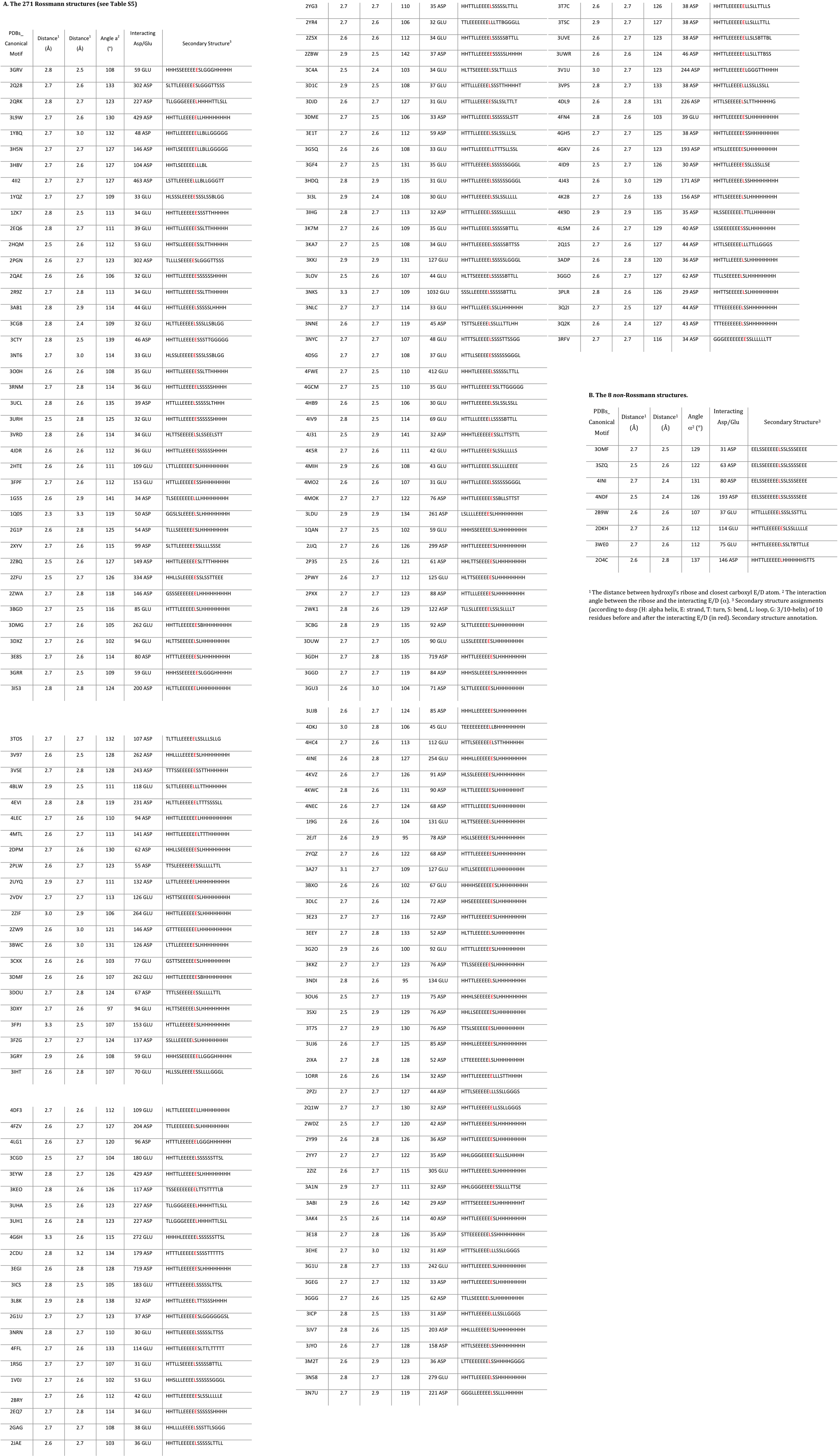

Supplement: S6 Table — (PNG) [file pbio.1002396.s023.png]

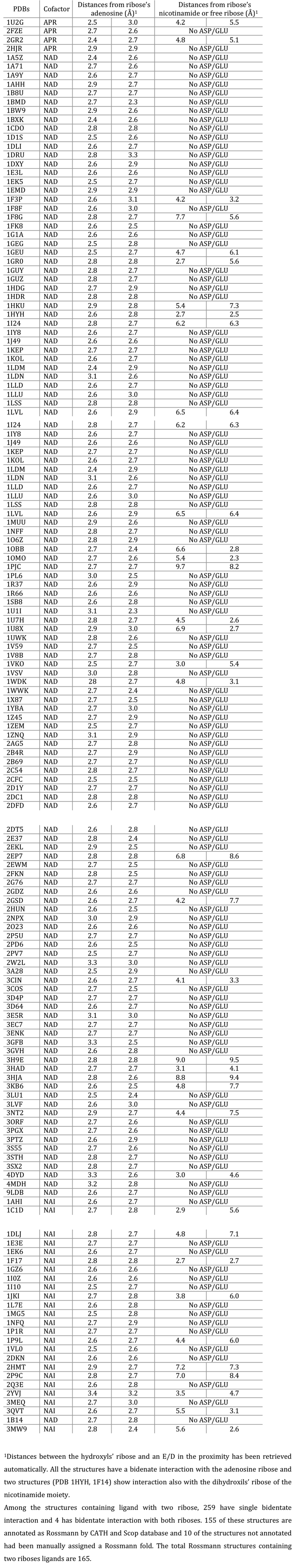

Supplement: S7 Table — Noted are the distances of the interacting E/D at the top of the β2 strand to the ribose hydroxyls. In all these cases, the interacting ribose belongs to the adenosine moiety of these cofactors and not to the nicotinamide (NAD, or NADH—annotated as NAI) or the free ribose (in APR). (PNG) [file pbio.1002396.s024.png]

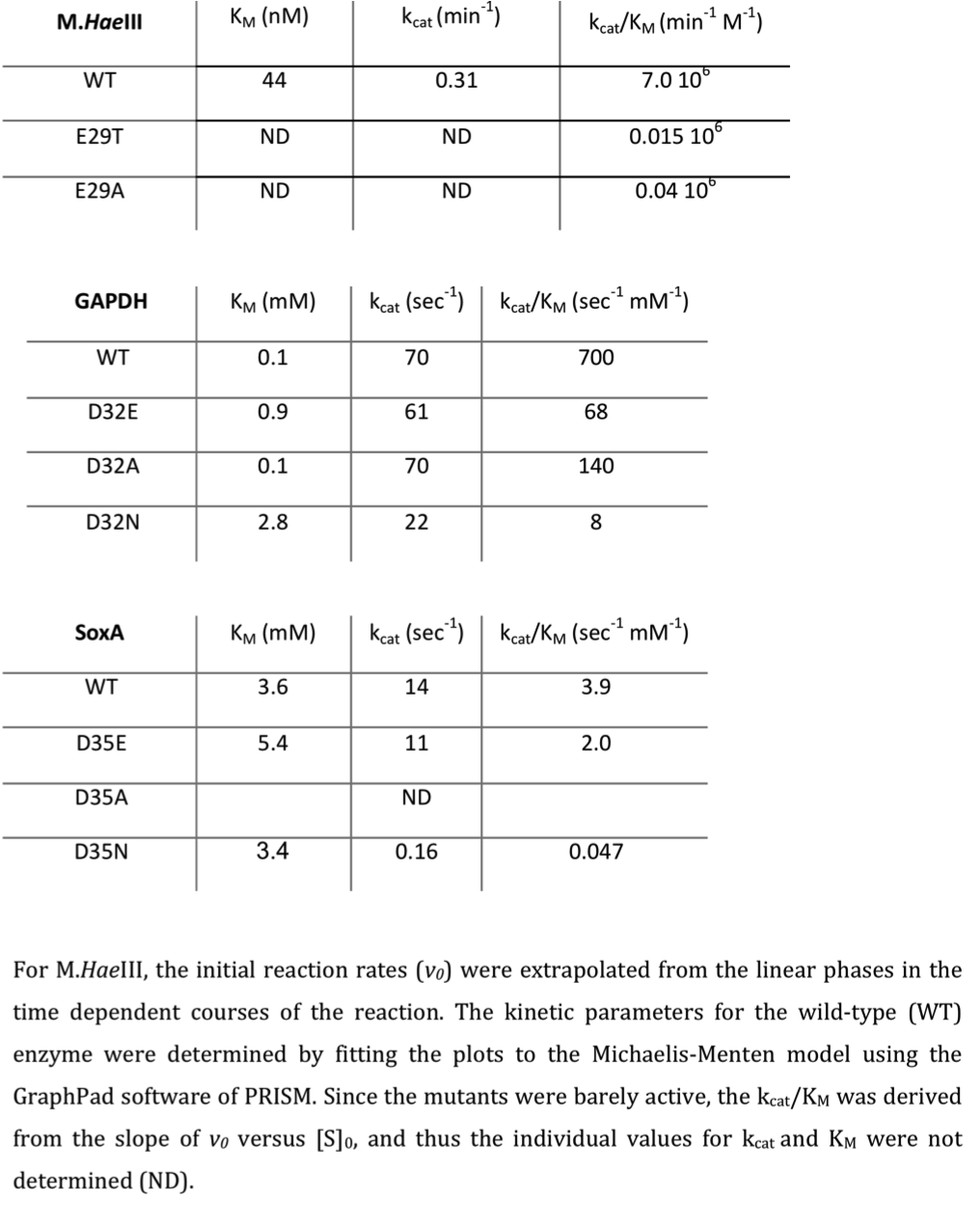

Supplement: S8 Table — (PNG) [file pbio.1002396.s025.png]

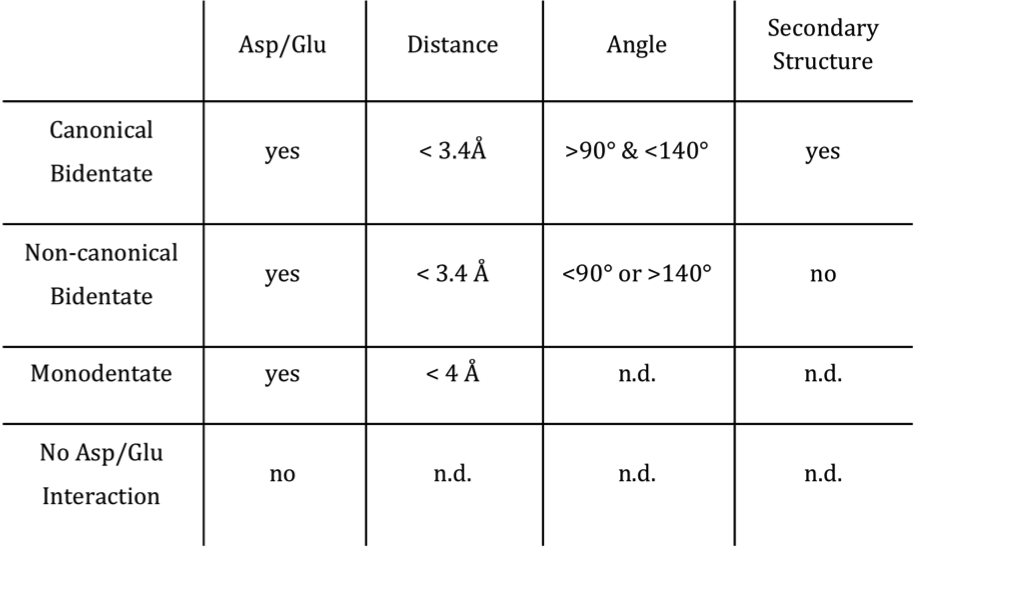

Supplement: S9 Table — (PNG) [file pbio.1002396.s026.png]
