# Supplementary material for: An Ancient Fingerprint Indicates the Common Ancestry of Rossmann-Fold Enzymes Utilizing Different Ribose-Based Cofactors
Source: PLoS Biol. 2016 Mar 3;14(3):e1002396. doi: 10.1371/journal.pbio.1002396 (PMC4777477; doi:10.1371/journal.pbio.1002396)
Supplement: S1 Text — (DOCX) [file pbio.1002396.s027.docx]

**Supporting Item**

**Quantum Mechanical Calculations**

## S1.1. Molecular Models

## S1.2. Conformational study of the ribose ring

## S1.3. Relative disposition of the dicarboxylate respect to the adenosine ring

## S1.4. Analysis of the PDB structures

## S1.5. Conformational search and scan along the angle α for model M1

**S1.6. Algorithm to optimize a molecular geometry by fixing the angle α, which is defined by four atoms**

**S1.7. Conformational analysis of the adenosine**

**S1.8. Study of the bidentate interaction by using model M2**

This section is divided into four subsections. Section S1.1 shows the model that was used to study the bidentate interaction by electronic structures calculations in solvent. In Section S1.2, we discuss the conformational analysis of the ribose ring. Section S1.3 defines the angle between the ribose and the carboxylate; and Section 1.4, contains the analysis of the interaction in various enzymes as well as the energetic and geometrical study of the factors that control the value of the angle of the bidentate interaction, i.e. the factors that deal with the canonical Rossmann interaction occur only in certain values of the angle. Last Section, Section 1.5 presents the algorithm that has been developed to optimize a structure by fixing the value of the angle that is defined as the angle between two bond vectors. Note that this algorithm is an original contribution since standard optimization procedures allow constraints of bond distances, bond angles, or dihedral angle, but here we have the angle between two bond vectors that do not share a common atom; so a new algorithm was required.

All electronic structure calculations in this study have been carried out by using the M06-2X/6-31+G(d,p)[1]^,^ [2] model chemistry including the effect of aqueous solvent by using the SMD solvation model [3]. We have computed all frequencies for all optimized structures to warranty that they correspond to minima on the potential energy surface (PES).

## S1.1. Molecular Models

In order to study the bidentate interaction between a carboxylate side-chain and the ribose’s 2´ and 3´ hydroxyls, we propose the model M1 shown in Scheme S1 that consists in a molecule of adenosine (that models the cofactor), and a molecule of acetate (that models the side chain).

Scheme S1. Model M1 used to study the bidentate interaction.

We characterized the bidentate interaction between the 2’ and 3’ hydroxyls of ribose and a carboxylate side chain by defining the angle α**,** and here we used quantum mechanical calculations to find the values that α takes for the lowest energy structures.  We defined **the interaction angle (α)** by the vectors (see.Fig S14):

**v1**: from CH_2_ to CO

**v2**: from C2R to C1R.

where the value of **α** is given by the equation:

## S1.2. Conformational study of the ribose ring

The five-membered ring of the adenoribose can exist in either envelope (E) or twist (T) conformations (see **Scheme S2**). Envelope conformations have one atom out of the plane of the ring formed by the four other ring atoms, while twist conformations have one atom above or below the plane of the ring formed by the three other ring atoms. The index atom that is above the plane is denoted as superscript, while a subscript denotes the index of the atom that is below the atom ring, i.e. ^2^E and E_2_ denote the envelope conformation with atom C2 above and below the ring plane respectively.

Scheme S2. Twist and envelope furanose ring conformations, where four atoms are in a plane.

## S1.3. Relative disposition of the dicarboxylate respect to the adenosine ring

The hydrogens of the OH groups in carbons C2R and C3R of the adenosine can adopt different orientations that lead to a double hydrogen bond with the dicarboxylate. Here we differentiate two different dispositions, as shown in Scheme S3, where endo and exo refer to both the direction of hydrogens in the 2´ and 3´ hydroxylic groups of the adenosine and the disposition of the dicarboxylate (see **Fig 2b**). Note that we use the endo/exo notation for the relative orientation of both molecules in similar sense that it is used in discussions of the stereoselectivity in Diels*–*Alder reactions [4].

Scheme S3. Schematic representation of the different dispositions of the OH groups in carbons C2R and C3R of the adenosine.

The value of α defines the relative orientation of the adenosine molecule with respect to the dicarboxylate, so low and high values of α are directly related to exo and endo orientations, respectively, and with non-canonical and canonical interactions respectively. As shown below, all PDB structures can be categorized by using endo and exo terminology.


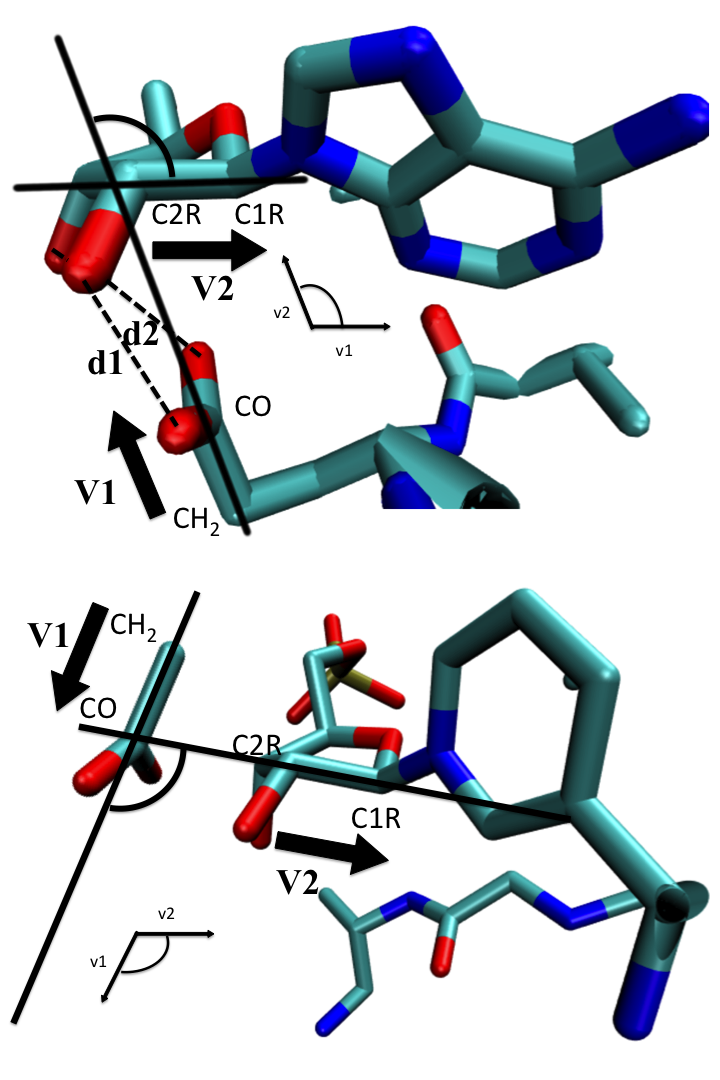


**a**

**b**

Fig S14. Disposition of the dicarboxylate with respect to the ribose ring. *a.* Endo bidentate interaction. *b.* Exo bidentate interaction.

## S1.4. Analysis of the PDB structures

We have analyzed the conformations of the ribose ring for an exhaustive list of PDB structures. Each structure has been characterized by the interatomic distances d1 and d2 the angle α (**Fig 2b** and **Fig S14** the endo/exo disposition of the dicarboxylate with respect to the ribose ring (see **Scheme S3**). The endo and exo classification of the structures was made by visual analysis of each structure.

Table S10: Angle α (in degrees) and atomic distances d1, and d2 (in Å) as defined in Fig. 2b for all chains in representative PDB structures. The bidentate interaction is characterized by the PDB label of the C2R atom in ribose and the conformation of the ribose ring.

| **PDB** | **d1 (Å)** | **Avarage (Å)** | **Standard Dev** | **d2 (Å)** | **Avarage (Å)** | **Standard Dev** | **Angle α (°)** | **Avarage (°)** | **Standard Dev** | **C2R atom in PDB** |
| --- | --- | --- | --- | --- | --- | --- | --- | --- | --- | --- |
| 1AHH | 2.9 | 3.05 | 0.212132 | 2.7 | 2.8 | 0.095742711 | 133.601 | 134.0615 | 0.651245 | HETATM 3730 C2B NAD A 256 54.445 14.842 7.147 1.00 35.98 C |
|  | 3.2 |  |  | 2.9 |  |  | 134.522 |  |  | HETATM 3774 C2B NAD B 256 28.161 8.477 -25.097 1.00 34.23 C |
| 1BWC | 2.9 |  |  | 2.8 |  |  | 114.891 |  |  | HETATM 3515 C2B FAD A 479 26.834 51.422 23.360 1.00 12.43 C |
| 1EG2 | 2.7 |  |  | 2.7 |  |  | 126.528 |  |  | HETATM 2231 C2' MTA A 401 28.724 23.610 -5.450 0.50 34.32 C |
| 1GEG | 2.5 | 2.5875 | 0.064087 | 2.8 | 2.8125 | 0.064086994 | 119.098 | 119.9354 | 0.779953 | HETATM14935 C2B NAD A2001 29.162 -16.748 -3.888 1.00 14.49 C |
|  | 2.5 |  |  | 2.9 |  |  | 118.959 |  |  | HETATM14996 C2B NAD B2002 30.000 16.099 16.900 1.00 12.51 C |
|  | 2.6 |  |  | 2.8 |  |  | 119.854 |  |  | HETATM15056 C2B NAD C 305 -18.161 1.210 25.834 1.00 19.11 C |
|  | 2.6 |  |  | 2.7 |  |  | 120.494 |  |  | HETATM15116 C2B NAD D 306 -17.735 1.321 -13.307 1.00 11.30 C |
|  | 2.7 |  |  | 2.9 |  |  | 121.267 |  |  | HETATM15116 C2B NAD D 306 -17.735 1.321 -13.307 1.00 11.30 C |
|  | 2.6 |  |  | 2.8 |  |  | 119.372 |  |  | HETATM15236 C2B NAD F 308 -31.117 -26.049 75.775 1.00 11.40 C |
|  | 2.6 |  |  | 2.8 |  |  | 120.348 |  |  | HETATM15297 C2B NAD G2003 15.866 -8.477 66.431 1.00 12.63 C |
|  | 2.6 |  |  | 2.8 |  |  | 120.091 |  |  | HETATM15358 C2B NAD H2004 15.841 -40.962 44.417 1.00 10.81 C |
| 1GZ6 | 2.6 | 2.725 | 0.386221 | 2.6 | 2.7 | 0.089973541 | 122.768 | 125.5918 | 5.119721 | HETATM 8972 C2B NAI A1306 36.192 46.141 34.740 1.00 23.23 C |
|  | 2.5 |  |  | 2.8 |  |  | 124.859 |  |  | HETATM 9016 C2B NAI B1304 0.334 52.636 46.572 1.00 41.29 C |
|  | 3.3 |  |  | 2.8 |  |  | 133.018 |  |  | HETATM 9060 C2B NAI C1304 -2.962 32.624 90.178 1.00 41.67 C |
|  | 2.5 |  |  | 2.6 |  |  | 121.722 |  |  | HETATM 9114 C2B NAI D1305 30.750 39.211 107.557 1.00 25.12 C |
| 1JG2 | 2.7 |  |  | 2.8 |  |  | 111.335 |  |  | HETATM 1706 C2' ADN A 500 25.807 45.925 17.075 1.00 19.33 C |
| 1V5E | 2.8 |  |  | 2.7 |  |  | 131.508 |  |  | HETATM 4629 C2B FAD A1601 30.765 24.921 8.139 1.00 9.05 C |
| 2A14 | 2.6 |  |  | 2.7 |  |  | 102.591 |  |  | HETATM 2000 C2' SAH A4001 24.072 45.450 6.781 1.00 9.22 C |
| 2AVD | 2.6 | 2.65 | 0.070711 | 2.6 | 2.65 | 0.057735027 | 111.589 | 112.0665 | 0.675287 | HETATM 3427 C2' SAM A 501 2.902 6.181 18.103 1.00 15.63 C |
|  | 2.7 |  |  | 2.7 |  |  | 112.544 |  |  | HETATM 3454 C2' SAM B 601 -4.089 -8.740 64.181 1.00 18.45 C |
| 2GR2 | 5.6 |  |  | 2.7 |  |  | 103.768 |  |  | HETATM 3001 C2B FAD A1449 59.687 12.204 4.076 1.00 11.94 C |
| 2HMU | 2.7 | 2.7 | 0 | 2.8 | 2.8 | 0 | 128.629 | 130.2455 | 2.286076 | HETATM 2165 C2' ATP A 601 111.328 53.585 13.366 1.00 40.58 C |
|  | 2.7 |  |  | 2.8 |  |  | 131.862 |  |  | HETATM 2196 C2' ATP B 602 118.874 43.127 37.467 1.00 41.99 C |
| 2PBF | 2.4 | 2.5 | 0.141421 | 2.8 | 2.8 | 0 | 105.557 | 105.341 | 0.30547 | HETATM 3492 C2' SAH A 301 33.602 -4.726 -7.824 1.00 30.61 C |
|  | 2.6 |  |  | 2.8 |  |  | 105.125 |  |  | HETATM 3518 C2' SAH B 301 33.493 4.719 -26.696 1.00 29.43 C |
| 2XXB | 2.7 | 2.7 | 0 | 2.8 | 2.8 | 0 | 127.078 | 123.347 | 5.276431 | HETATM 4642 C2' AMP A1332 1.759 -29.302 -4.715 1.00 26.41 C |
|  | 2.7 |  |  | 2.8 |  |  | 119.616 |  |  | HETATM 4665 C2' AMP B1332 -21.068 -34.656 -12.491 1.00 15.70 C |
| 3GVI | 2.5 | 2.65 | 0.104881 | 2.8 | 2.7 | 0.154919334 | 116.56 | 117.7685 | 1.65064 | HETATM13731 C2' ADP A 401 36.827 22.239 45.874 1.00 18.55 C |
|  | 2.7 |  |  | 2.8 |  |  | 120.262 |  |  | HETATM13758 C2' ADP B 401 9.600 74.734 45.640 1.00 19.91 C |
|  | 2.6 |  |  | 2.4 |  |  | 116.091 |  |  | HETATM13785 C2' ADP C 401 5.606 71.953 22.434 1.00 19.74 C |
|  | 2.7 |  |  | 2.8 |  |  | 116.56 |  |  | HETATM13812 C2' ADP D 401 41.029 24.698 22.657 1.00 21.67 C |
|  | 2.8 |  |  | 2.7 |  |  | 119.01 |  |  | HETATM13839 C2' ADP E 401 24.208 97.647 -11.221 1.00 21.47 C |
|  | 2.6 |  |  | 2.7 |  |  | 118.128 |  |  | HETATM13866 C2' ADP F 401 82.725 91.447 11.925 1.00 22.01 C |
| 1GUY | 2.8 | 2.75 | 0.070711 | 2.7 | 2.65 | 0.1 | 121.128 | 121.674 | 0.772161 | HETATM 4430 C2B NAD A1307 63.118 69.047 5.745 1.00 28.49 C |
|  | 2.7 |  |  | 2.6 |  |  | 122.22 |  |  | HETATM 4477 C2B NAD C1307 93.895 19.123 29.309 1.00 24.84 C |
| 1SB8 | 2.6 |  |  | 2.8 |  |  | 125.972 |  |  | HETATM 2673 C2B NAD A 342 53.988 -3.035 86.564 1.00 14.22 C |
| 2FKN | 2.5 | 2.65 | 0.129099 | 2.8 | 2.8 | 0 | 95.89 | 98.8975 | 3.290022 | HETATM16816 C2B NAD A5555 -2.672 0.122 52.229 1.00 19.81 C |
|  | 2.8 |  |  | 2.8 |  |  | 102.698 |  |  | HETATM16864 C2B NAD B6555 -2.923 -0.428 -2.608 1.00 24.33 C |
|  | 2.6 |  |  | 2.8 |  |  | 100.583 |  |  | HETATM16912 C2B NAD C7555 45.799 32.030 -2.785 1.00 20.62 C |
|  | 2.7 |  |  | 2.8 |  |  | 96.419 |  |  | HETATM16960 C2B NAD D8555 43.326 31.828 51.729 1.00 28.46 C |
| 1LDM | 2.9 |  |  | 2.4 |  |  | 108.562 |  |  | HETATM 2553 C2B NAD A 330 2.231 28.596 12.752 1.00 19.93 C |
| 1P1R | 2.7 | 2.65 | 0.057735 | 2.7 | 2.7 | 0 | 131.315 | 129.2285 | 1.69348 | HETATM11156 C2B NAI A 377 33.518 5.369 3.520 1.00 16.83 C |
|  | 2.6 |  |  | 2.7 |  |  | 127.167 |  |  | HETATM11212 C2B NAI B 377 13.040 35.129 31.333 1.00 14.77 C |
|  | 2.7 |  |  | 2.7 |  |  | 129.211 |  |  | HETATM11276 C2B NAI C 377 47.360 5.009 46.413 1.00 13.25 C |
|  | 2.6 |  |  | 2.7 |  |  | 129.221 |  |  | HETATM11332 C2B NAI D 377 24.768 35.476 71.563 1.00 16.70 C |
| 3TNL | 2.6 | 2.6 | 0 | 5.5 | 5.45 | 0.057735027 | 67.636 | 68.24575 | 1.020464 | HETATM 9410 C2B NAD A 292 20.106 44.147 55.198 1.00 17.82 C |
|  | 2.6 |  |  | 5.4 |  |  | 69.315 |  |  | HETATM 9467 C2B NAD B 292 43.217 38.145 115.207 1.00 15.14 C |
|  | 2.6 |  |  | 5.5 |  |  | 67.149 |  |  | HETATM 9512 C2B NAD C 292 7.041 23.186 4.554 1.00 18.09 C |
|  | 2.6 |  |  | 5.4 |  |  | 68.883 |  |  | HETATM 9569 C2B NAD D 292 -16.092 29.380 64.604 1.00 16.21 C |
| 3B4W | 2.7 |  |  | 2.6 |  |  | 17.99 |  |  | HETATM 3621 C2D NAD A 500 81.815 26.979 23.249 1.00 26.26 C |
| 3C7A | 3 |  |  | 3.1 |  |  | 23.274 |  |  | HETATM 3104 C2B NAD A 405 95.167 34.941 52.412 1.00 19.89 C |
| 3GD4 | 2.5 | 2.5 | 0 | 2.9 | 2.85 | 0.070710678 | 12.661 | 16.237 | 5.057228 | HETATM 6868 C2B NAD A 700 -35.935 31.840 -17.179 1.00 47.05 C |
|  | 2.5 |  |  | 2.8 |  |  | 19.813 |  |  | HETATM 6965 C2B NAD B 700 -54.051 34.649 -57.937 1.00 52.32 C |
| 3JU8 | 2.3 |  |  | 5.1 |  |  | 24.926 |  |  | HETATM 7739 C2B NAD A 501 16.464 32.693 58.230 0.69 42.75 C |

Table S11: Distribution of the ribose ring conformation and the relative endo/exo disposition between the ribose ring and the carboxylate.

|  | Endo | Exo | E_1_/^2^E | Other envelope conformations |
| --- | --- | --- | --- | --- |
| Rossmann-canonical  (*n* = 263) | 100% | 0% | 96% | 4% |
| Rossmann-*non*-canonical (*n* = 38) | 29% | 71% | 47% | 53% |

The distributions for the two main groups of the PDB structures (Rossmann-canonical and the Rossmann-non-canonical) with respect to the disposition of the carboxylate relative to the adenosine (exo or end) and the conformation of the furanose ring. All the Rossmann canonical interactions occur with the endo disposition (100% of 263 structures analyzed) and the ribose ring conformation is almost completely restricted mainly to E_1_ and ^2^E conformations (96% of 263 structures analyzed), i.e. the proteins in this group have very similar conformational characteristics).

## S1.5. Conformational search and scan along the angle α for model M1

We carried out an exhaustive conformational search for model M1 on the potential energy surface (PES). Two different techniques were considered to generate the initial Cartesian coordinates of the structures that are energetically minimized.

In a first approximation we considered the Cartesian coordinates of various PDB structures. **Table S12** shows the conformations, the values of the angle α, and the relative energies (where the zero of energy is set at the lowest energy optimized structure) of all the optimized structures. Note that some PDB structures lead to the same optimized structure of model M1.

A second technique was used to explore more widely the coordinate space of the system (**Table S13**) by combining different ribose ring configurations (shown in **Scheme S2**) with endo, exo configurations (**Scheme S3**).

Table S12. Comparison of the ribose ring conformation and values of angle in the PDB structures and in the energy minimized structures using model M1. The initial Cartesian coordinates for each optimized conformation was taken from their counterparts in the PDB structure.

| **PDB** | **Conformation** | **Angle α (°)** | **Model M1** | | |
| --- | --- | --- | --- | --- | --- |
|  |  |  | **Conformation** | **Angle α (°)** | **E(kcal/mol)** |
| 1guy | E_1_-endo | 121.128 | E_3_-endo | 133.590 | 0.84 |
| 1sb8 | E_1_-endo | 125.972 | E_3_-endo | 133.597 | 0.84 |
| 2fkn | E_1_-endo | 95.890 | E_3_-endo | 133.608 | 0.84 |
| 1ldm | ^2^E-endo | 108.562 | ^2^E-endo | 132.272 | 0.00 |
| 1p1r | E_1_-endo | 131.315 | E_1_-endo | 130.711 | 1.46 |
| 3tnl | ^2^E-exo | 67.636 | ^a^ |  |  |
| 3b4w | ^2^E-exo | 17.990 | ^2^E-exo | 14.451 | ^b^ |
| 3c7a | ^3^E-exo | 23.274 | E_3_-exo | 33.725 | 1.04 |
| 3gd4 | E_1_-exo | 12.661 | ^3^E-exo | 37.071 | 2.35 |
| 3ju8 | E_3_-exo | 24.926 | ^2^E-exo | 42.823 | 1.89 |
| 1ahh | ^2^E-endo | 133.601 | E_3_-endo | 133.583 | 0.84 |
| 1bwc | E_1_-endo | 114.891 | E_1_-endo | 130.698 | 1.46 |
| 1eg2 | E_1_-endo | 126.528 | E_3_-endo | 100.663 | 0.34 |
| 1geg | E_1_-endo | 119.098 | E_3_-endo | 133.607 | 0.84 |
| 1gz6 | E_1_-endo | 122.768 | E_3_-endo | 132.861 | 0.42 |
| 1jg2 | E_1_-endo | 111.335 | E_1_-endo | 130.383 | 1.45 |
| 1v5e | ^2^E-endo | 131.508 | E_3_-endo | 133.619 | 0.84 |
| 2a14 | E_1_-endo | 102.591 | E_3_-endo | 97.233 | 0.41 |
| 2avd | E_1_-endo | 111.589 | E_3_-endo | 101.845 | 0.34 |
| 2gr2 | E_1_-endo | 103.768 | ^2^E-endo | 130.154 | 4.57 |
| 2hmu | ^2^E-endo | 128.629 | E_3_-endo | 128.710 | 0.50 |
| 2pbf | E_1_-endo | 105.557 | E_3_-endo | 101.820 | 0.34 |
| 2xxb | E_1_-endo | 127.078 | E_1_-endo | 130.735 | 1.46 |
| 3gvi | ^3^E-endo | 116.560 | E_1_-endo | 130.391 | 1.45 |

^a)^ Structure not found.

^b)^ The PDB has no adenine substituent, and its corresponding model M1 has as different number of atoms than the rest of structures for model M1.

**Table S13. Relative energies (in kcal/mol) and angle α (in degrees) for various optimized conformers of model M1.**

| **Conformation** | **Angle α (°)** | **E(kcal/mol)** |
| --- | --- | --- |
| E_3_-endo | 133.590 | 0.84 |
| **^2^E-endo** | **132.272** | **0.00** |
| E_1_-endo | 130.711 | 1.46 |
| E_3_-exo | 33.725 | 1.04 |
| ^3^E-exo | 37.071 | 2.35 |
| ^2^E-exo | 42.823 | 1.89 |
| E_3_-endo | 100.663 | 0.34 |
| E_3_-endo | 132.861 | 0.42 |
| E_3_-endo | 97.233 | 0.41 |
| ^2^E-endo | 130.154 | 4.57 |
| E_3_-endo | 128.710 | 0.50 |
| ^3^E-exo | 37.600 | 1.01 |
| ^1^E-endo | 101.326 | 0.56 |
| ^1^E-exo | 29.233 | 1.43 |
| ^2^E-endo | 81.497 | 1.37 |
| E^1^-endo | 131.15 | 0.67 |
| E^1^-endo | 129.261 | 0.69 |
| E^1^-endo | 129.779 | 0.67 |
| ^2^T3-exo | 14.203 | 1.16 |
| ^3^T_2_-endo | 160.396 | 5.87 |
| ^3^E-endo | 96.017 | 1.22 |
| ^3^E-exo | 30.660 | 1.58 |
| E_1_-endo | 118.745 | 5.94 |
| ^2^E-endo | 131.744 | 0.46 |
| E_4_-exo | 18.928 | 1.16 |
| ^2^T_3_-endo | 134.358 | 10.25 |
| ^2^E-exo | 9.888 | 1.20 |
| ^3^E-endo | 91.347 | 0.81 |
| ^3^E-exo | 34.639 | 1.02 |
| ^3^E-exo | 52.411 | 1.97 |
| ^2^E-exo | 80.041 | 1.37 |
| ^3^E-endo | 91.315 | 0.81 |
| **^3^E-exo** | **36.697** | **1.00** |
| E_3_-endo | 130.477 | 0.51 |
| ^2^E-exo | 14.387 | 1.14 |
| E_4_-endo | 108.009 | 1.30 |
| E_4_-exo | 34.639 | 1.65 |
| ^1^E-endo | 101.334 | 0.56 |
| ^1^E-exo | 31.589 | 1.43 |
| E_1_-endo | 130.414 | 0.68 |
| ^2^E-exo | 18.673 | 1.18 |
| E_3_-endo | 130.142 | 0.93 |
| ^2^T_3_-endo | 80.841 | 1.82 |

All optimized structure of model M1 appear in Tables S12 and S13. As can be seen in the tables, the lowest-energy conformation corresponds to an endo configuration with α equal to 132 degrees; while the lowest-energy exo conformation (that shows an angle α of 37 degrees) is 1 kcal/mol higher in energy (data highlighted in Table S13). It is well known (for instance in the Diels-Alder reactions) that the endo interactions are often more favorable than the exo interactions.

A relaxed potential energy surface scan along the coordinate defined by the angle α has been performed starting from the lowest energy conformations, ^2^E-endo and ^3^E-exo (see **Table S12**). In the scan all degrees of freedom have been optimized with the exception of α. As explained below a new algorithm has been implemented to perform the calculations.

As shown in **Fig S15** the energetic profile of the endo and exo configurations show one minimum at 132 and 37 degrees respectively, both minima correspond to ^2^E-endo and ^3^E-exo structures that have been completely optimized previously (**Table S13**), i.e. they correspond to stable structures on the potential energy surface.

From our calculations by using model M1 we obtained two lowest energy minima for both endo and exo conformations (**Fig S14**), ^2^E-endo and ^3^E-exo, where ^2^E/^3^E denotes the envelop form of the ribose ring, and endo or exo define the relative orientation of the ribose with respect to the carboxylate. The lowest energy structure, ^2^E-endo, exhibits an angle α of ~130° that overlaps the canonical Rossmann motif. Beyond the overlap of the interaction angle, they match on the ribose ring conformation. Accordingly, the vast majority of Rossman enzymes that posses the canonical motif have the ribose in E_1_ or ^2^E (96% of 263 structures adopt this configuration; **Table S16**). The other minimum, ^3^E-exo correspond to an angle range of ~37° (**Table S15**) that seems to dominate the *non*-canonical interactions that occur in the range of ~10°, and where the furanose configuration ^3^E seems as favorable as E_1_ and ^2^E.

Energetically, ^2^E-endo and ^3^E-exo are only different by 1.0 kcal/mol (note that 0.5 kcal/mol corresponds to <3-fold lower affinity). All energy optimizations initiated from canonical Rossmann PDB structures converged to the endo configuration, with the ribose primarily in E_1_ and ^2^E and α ≈ 130°. In contrast, minimization energy calculations initiated from *non*-canonical PDB structures converged to the exo configuration, with the ribose primarily in ^2^E and ^3^E and α ≈ 14–43°. Interconversion between the two configurations, endo and exo, was not observed during the optimization of the geometry, because it involves a major change in the geometry overcoming high energy barriers.

To study the bidentate interaction between a carboxylate side-chain and the ribose’s 2´ and 3´ hydroxyls, we carried out quantum mechanics calculations on model M1 (shown in Scheme S1) that consists in a molecule of adenosine (that models the cofactor), and a molecule of acetate (that models the side chain).

All electronic structure calculations in this study have been carried out by using the M06-2X/6-31+G(d,p)[1, 2] model chemistry including the effect of aqueous solvent by using the SMD solvation model [3].

We performed an exhaustive conformational search for model M1. Starting from the lowest energy optimized structures obtained of model M1, ^2^E-endo and ^3^E-exo, we carried out a relaxed potential energy surface scan along the coordinate defined by α (see **Fig S14**). In the scan all degrees of freedom have been optimized with the exception of the angle α. This was accomplished by interfacing the *Gaussian 09*[5] with a utility program we wrote that allows a constraint on the angle between two vectors.

**S1.6. Algorithm to optimize a molecular geometry by fixing the angle α, which is defined by four atoms**

The optimization of the molecular structure was accomplished by using the Berny algorithm[6] as implemented in Gaussian where the initial geometry is modified to define the angle α as the angle defined by three atoms. During the optimization the energy, the gradient and the Hessian was computed on the correct geometry by using an external code that we wrote. See **Scheme S4** and **Scheme S5**.

**Scheme S4. Flowchart of the algorithm to optimize the M1 model freezing the value of the angle α defined by the atoms A-B-C-D**.

**Scheme S5. Details about the change in internal coordinates for the translation step during the optimization.**

## S1.7. Conformational analysis of the adenosine

Since the disposition of the hydrogen in hydroxyls 2’ and 3’ in the ribose ring is important to understand the bidentate interaction, we carried out a conformational study that takes into account the rotation of the OHs around the C-O bonds. Considering that there are three minima for rotation of each OH, ideally 3^2^ = 9 hydroxyl rotamers can be formed. Here we denoted all rotamers by using the notation gauche (g), anti-gauche (a), or trans (t)[7] for two OH groups, the 2’ and 3’ OH group respectively based on the values of the dihedral angle H-O-C2’/3’-H (see **Scheme S6**).

Scheme S6. Newman projections along the C2’-O’2 and C3’-O3’ bonds in the ribose ring. That show the different gauche, anti-gauche and trans disposition of the H in of the hydroxilic groups.


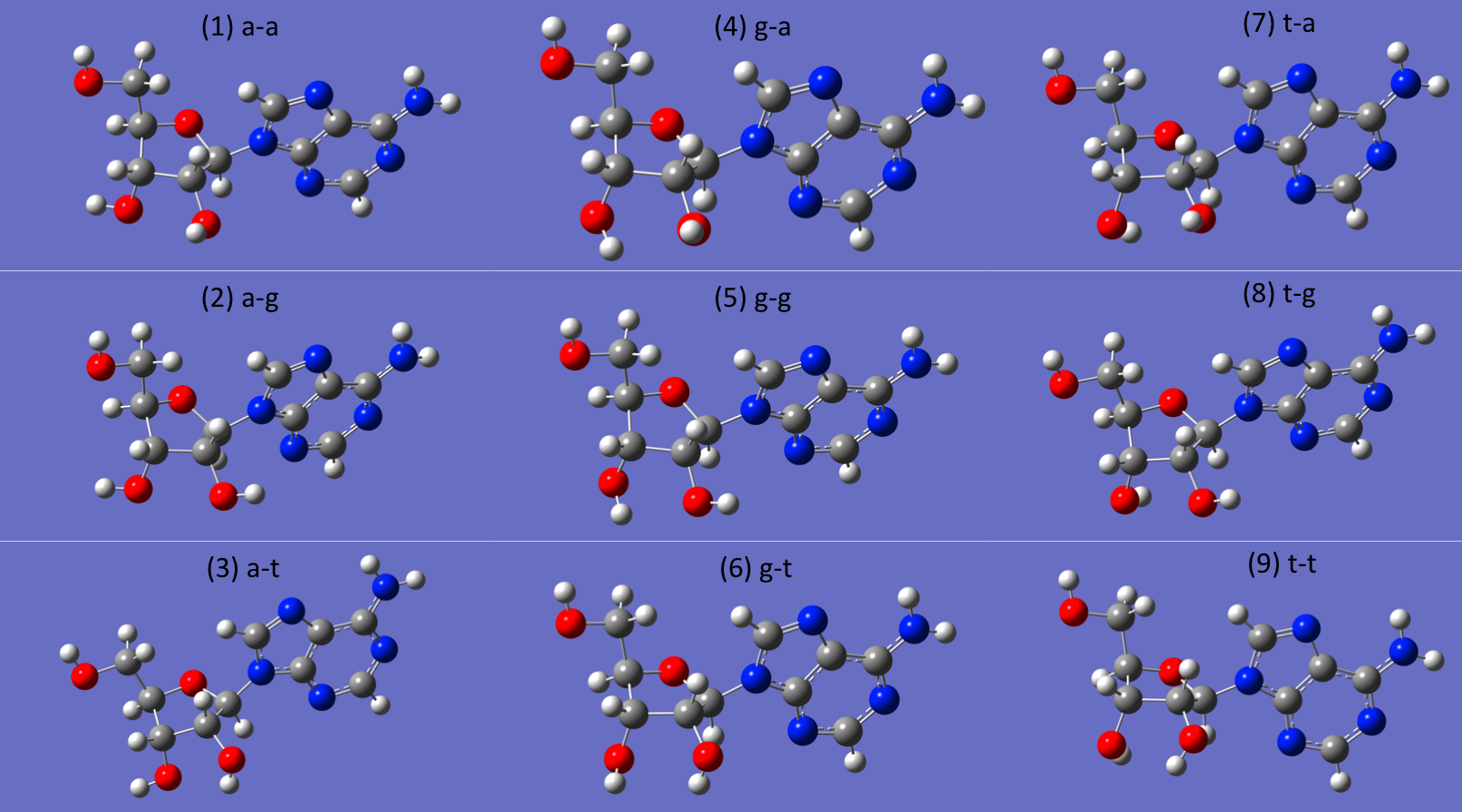


Fig S15: Structures of nine possible hydroxyl rotamers of adenosine.

After energy minimization we found nine optimized hydroxyl rotamers, shown in **Fig S15**. The ribose ring in all structures adopts a ^2^E envelope form. The relative energies (in kcal/mol) with respect to the lowest-energy structure and the H—H distance between the H of the 2’ and 3’ OH groups appear in **Table S14**. Note that our conformational analysis only considers the rotation of the 2’ and 3’ OH groups of the ribose ring but not other degrees of freedom of as the different conformation of the ribose ring and the rotation of the OH in the CH_2_OH functional group.

**Table S14. The relative energies (in kcal/mol) and H—H distance (in Å) between the H of the 2’ and 3’ OH groups for all nine optimized structures of adenosine.**

| Rotamers | E(kcal/mol) | R_H-H_(Å) |
| --- | --- | --- |
| a-a | 1.26 | 4.00 |
| a-g | 1.40 | 4.55 |
| a-t | 0.00 | 3.11 |
| **g-a** | **0.19** | **2.72** |
| g-g | 0.56 | 3.26 |
| **g-t** | **0.02** | **2.31** |
| t-a | 1.83 | 3.53 |
| t-g | 1.98 | 3.90 |
| **t-t** | **0.53** | **2.55** |

##

## S1.8. Study of the bidentate interaction by using model M2

In order to get the lowest energy structures for different values of α, here, we proposed a new approach. This new approach involves study the system with a new molecular model, called M2, and to freeze some degrees of freedom that entails freezing the value of α.

### S1.8.1. Model system

To reduce the computational cost of the calculations, for this part of the study, we carried out electronic structure calculations using model system M2, which consists a molecule of acetate and a simplified analogue of adenosine, where the adenine substituent and the -CH_2_OH functional groups have been replace by an -NH_2_ and a –CH_3_ group, respectively (see **Scheme S7**).

Scheme S7. Model M2 for studying the bidentate interaction.

### S1.8.2. Fully optimized structures of model M2

In the optimized structure of an isolated molecule of acetate, the O—O distance between the two atoms of oxygen in the carboxylic group is 2.23 Å. If we consider that two hydrogen bonds are established between the molecules of adenosine and acetate, the H—H distance between the two atoms of hydrogen of the OH groups in position 2’ and 3’ should not be much larger than that distance. By using this criterion we chose three hydroxyl rotamers, g-a, g-t, and t-t (they are highlighted in **Table S14**) that show the lowest O—O distances (2.72, 2.31 and 2.55 Å respectively). Using the geometrical parameters of these three structures we build their counterpart structures for model M2, that subsequently were used as starting geometries for energy minimization. By following this procedure we obtained three fully optimized structures, shown in **Fig S16** and whose Cartesian coordinates are given in Section 2.4. The ribose ring in t-t, g-t, and g-a structures adopts an E_1_, ^2^E, and ^2^E envelope form, respectively. According to the results of our electronic structure calculations the t-t conformation is the lowest energy structure, while the g-a and g-t conformations are 0.1 and 0.6 kcal/mol respectively higher in energy.

| 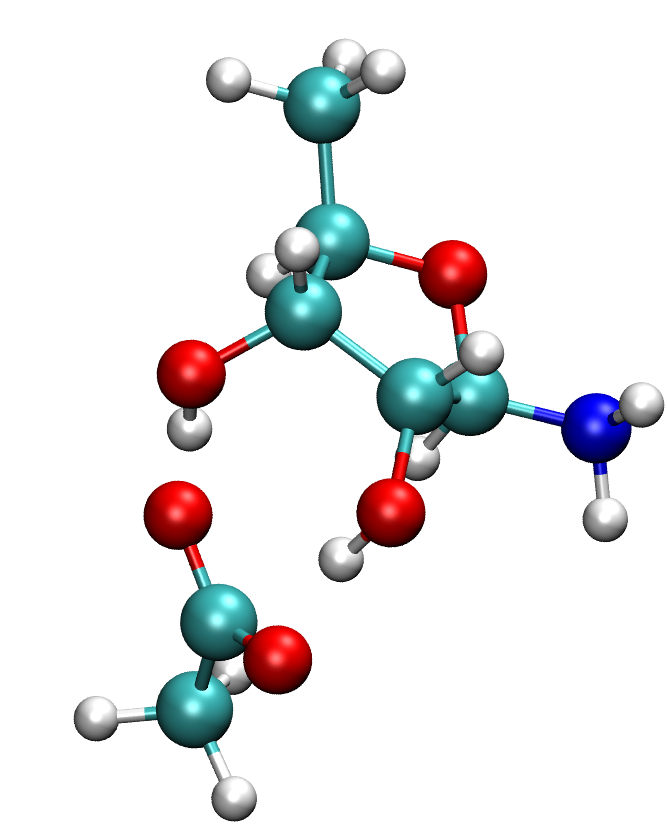 | 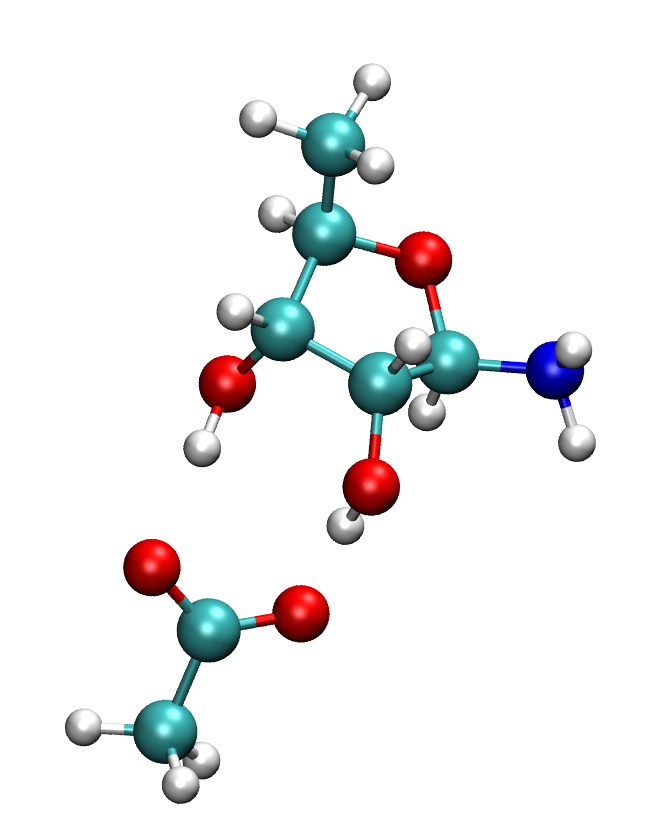 | 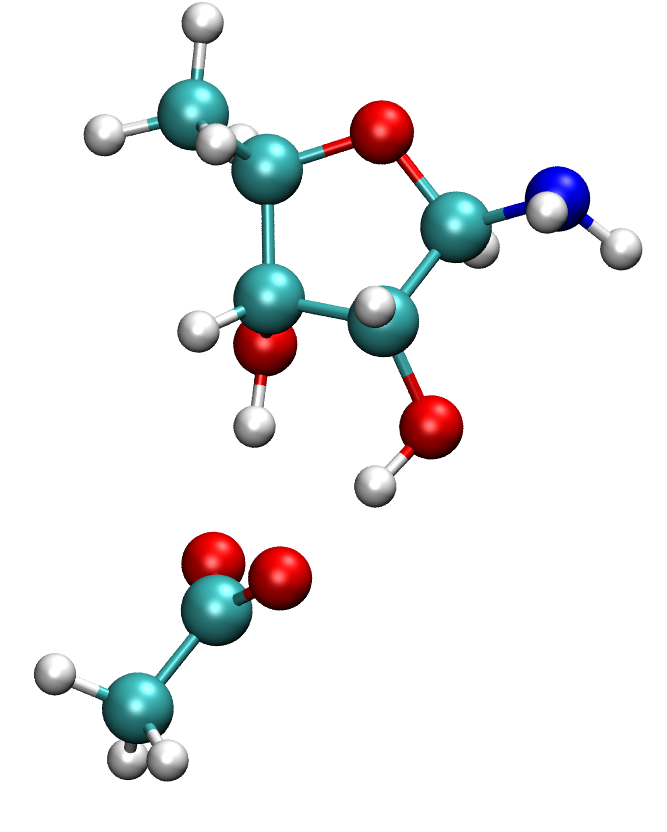 |
| --- | --- | --- |
| (a) t-t (E_1_) | (b) g-t (^2^E) | (c) g-a (^2^E) |

Fig S16: Fully optimized structures of model M2.

### S1.8.3. Partial optimization of M2

To locate the lowest energy structures for various values of α in a range of 0 to 180 degrees, we carried out partial optimizations of model M2, where the following degrees of freedom were fixed:

1. The angle defined by the carbon of the methyl group of the acetate, the carbon of the carboxylate group of the acetate and C2’ of the ribose ring.
2. The angle between the three points that are defined by the positions of the carbon in the COO^-^ functional group of the acetate, C2’ and C1’ of the ribose.
3. The dihedral angle defined by the following four atoms: the carbon of the CH_3_ group in the acetate, the carbon of the COO^-^ group in the acetate, C2’ of the ribose, and C1’ of the ribose.

These degrees of freedom are shown in **Scheme S8.**

Scheme S8. In red shows the degrees of freedom that have been frozen during the partial optimization of model M2.

Freezing the values of these three coordinates, the value of the angle α is also fixed, and the potential energy surface of the system can be explored by performing relaxed energy minimization scans along the three coordinates that have been mentioned before (the two angles and the dihedral angle). All geometry optimizations start from the three fully optimized structures described above, i.e. ‘t-t’, ‘g-t’ and ‘g-a’ and the values of the angles were frozen at ± 13^o^, ± 26^o^, and ± 39^o^ while the dihedral angle was frozen at values of ± 15^o^, ± 30^o^, and ± 45^o^. Of all the geometries, only ~500 of them were converged to optimized structures and the relative energies (with respect to the lowest-energy structure, the fully optimized ‘t-t’ structure) of the geometries within 5 kcal/mol and the angle α of each geometry are plotted in **Fig S17**. The profile with the relative energy of lowest-energy structure for each value is plotted in **Fig 4b**.


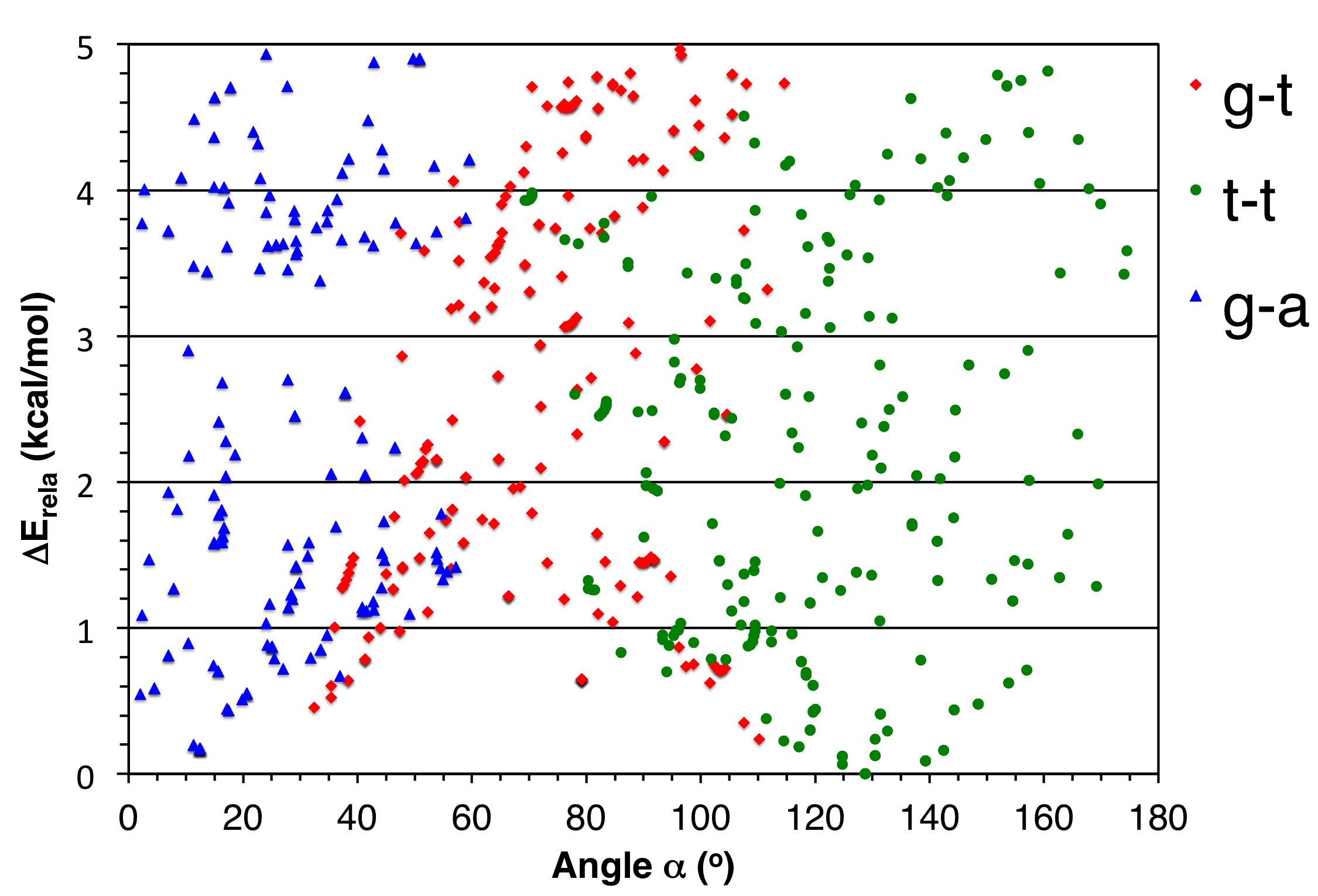


Fig S17. The relative energies (ΔE_rela_, in kcal/mol) of the structures of model M2 obtained by performing a relaxed energy minimized scan along the coordinates shown in Scheme S8. In red, green and blue all structures obtained from the relaxed optimized scan that start from the fully optimized structures g-t, t-t and g-a respectively.

## S1.9. Cartesian coordinates

### S1.9.1 Cartesian coordinates of the lowest-energy structures of model M1

**^2^E-endo**

C 1.069978 4.362967 -0.647158

C 1.636462 3.086384 -0.062231

O 2.332296 2.351596 -0.829032

O 1.372269 2.799651 1.140804

O 2.338699 -3.531905 -0.134651

C 2.960900 -2.749141 -1.148054

C 2.591557 -1.300355 -0.928148

O 1.169332 -1.157892 -1.141257

C 2.894695 -0.775233 0.492771

O 3.592375 0.451553 0.491137

C 1.492319 -0.668137 1.125601

O 1.354828 0.295360 2.138045

C 0.601109 -0.398524 -0.092358

N -0.779469 -0.791825 0.073700

C -1.248205 -2.040662 0.405289

N -2.555677 -2.116933 0.440142

C -2.974869 -0.840936 0.107941

C -4.253376 -0.261453 -0.025674

N -5.384395 -0.951968 0.184087

N -4.331924 1.036884 -0.375220

C -3.194054 1.717503 -0.578342

N -1.938782 1.289747 -0.478387

C -1.889632 -0.005898 -0.130228

H 2.600069 -4.454138 -0.254919

H 0.679595 5.019120 0.131051

H 0.255486 4.096652 -1.328884

H 1.834056 4.882084 -1.229771

H 2.623715 -3.066232 -2.141937

H 4.052424 -2.842343 -1.100224

H 3.121843 -0.682263 -1.661653

H 3.513927 -1.485471 1.045415

H 3.080004 1.146033 -0.013549

H 1.227629 -1.641711 1.551152

H 1.473739 1.198409 1.746009

H 0.592051 0.670518 -0.334007

H -0.569909 -2.860829 0.600092

H -6.278664 -0.497053 0.066817

H -3.321476 2.758601 -0.859548

H -5.352452 -1.928715 0.436898

**^3^E-exo**

C 1.810831 0.942411 -0.428258

C 1.091805 -0.029591 0.525273

C -0.061111 0.838420 1.041446

O 0.356848 2.184799 0.941424

C 1.646404 2.270889 0.305278

N -1.287861 0.677024 0.261558

C -2.286806 -0.232756 0.505808

C -3.184804 -0.078597 -0.544787

N -2.754151 0.894078 -1.426032

C -1.620462 1.307504 -0.910774

C -4.326390 -0.904450 -0.521956

N -4.462217 -1.779075 0.492396

C -3.508519 -1.821079 1.436117

N -2.397302 -1.096070 1.529588

O 1.907663 -0.368370 1.624265

N -5.263869 -0.861417 -1.483298

C 1.689528 3.476021 -0.597513

O 1.606989 4.647376 0.206527

O 3.171710 0.659004 -0.637740

H 1.668008 5.420042 -0.370020

H -6.110124 -1.402619 -1.375952

H -5.227522 -0.147031 -2.196038

H -3.672596 -2.545513 2.228152

H -0.990798 2.080483 -1.329505

H 1.260836 0.977887 -1.382043

H 0.720101 -0.927656 0.018223

H -0.305863 0.588298 2.076226

H 2.419155 2.359091 1.080286

H 2.633904 3.452550 -1.155835

H 0.856343 3.434622 -1.310450

H 2.524583 -1.087109 1.317146

H 3.253064 -0.301260 -0.912705

O 3.369225 -1.846441 -1.347763

C 3.524680 -2.689409 -0.412622

O 3.358424 -2.429627 0.814784

C 3.899883 -4.103905 -0.800063

H 4.391701 -4.620244 0.025150

H 2.980290 -4.644678 -1.047680

H 4.539633 -4.105330 -1.684112

### S1.9.2 Cartesian coordinates of the lowest-energy optimized structures of M2

1. **t-t**

C -3.09293500 -1.62358800 -0.65581400

C -1.76413900 -0.91738700 -0.51936500

O -1.84099300 0.37017700 -1.15102500

C -0.97969700 0.85796400 0.92940500

O 0.11863900 1.24440500 1.72112500

C -1.31221700 -0.65548400 0.93948400

O -0.22944600 -1.47553500 1.32928800

C -0.82164600 1.17894400 -0.56033400

N -1.00486100 2.54431700 -0.96150700

H -3.88042300 -1.05539500 -0.15059800

H -3.02789900 -2.61416800 -0.19559800

H -0.97008300 -1.49681500 -1.01411200

H -1.85282900 1.41549800 1.29092600

H 0.95139300 0.86678000 1.33796800

H -2.12385400 -0.86773600 1.64163400

H 0.52294900 -1.36688200 0.68489600

H 0.16382500 0.83924300 -0.91269700

H -3.36349100 -1.75001400 -1.70779100

H -0.11647500 3.03443700 -0.92606500

H -1.64557500 3.01642400 -0.32704500

C 3.88748500 -0.39554300 -1.03727100

H 4.54582100 0.43751400 -0.79030800

H 3.61297800 -0.34695500 -2.09480600

C 2.62982800 -0.37872400 -0.19313000

O 1.77890100 -1.29591000 -0.40047100

O 2.48829600 0.53451100 0.67034500

H 4.41442500 -1.34042700 -0.87781000

**(b) g-t**

C -3.45606300 -1.52059200 0.31041100

C -2.25133500 -1.04928300 -0.48223200

O -2.46603600 0.31639400 -0.91411000

C -0.91490300 0.41459700 0.80869000

O 0.32037500 0.88250600 1.29552500

C -0.94838500 -1.03556400 0.32242700

O 0.12541400 -1.31368000 -0.56079100

C -1.43544400 1.17460600 -0.41192800

N -1.98760400 2.48333800 -0.18427000

H -3.55304700 -0.95545300 1.24327700

H -3.35511900 -2.58270300 0.55223600

H -2.11689700 -1.66930400 -1.37542900

H -1.64762300 0.53094700 1.61792500

H 1.01594700 0.85189200 0.58765300

H -0.97127200 -1.75754700 1.14744300

H 0.99098800 -1.26608100 -0.08653400

H -0.63388700 1.27085600 -1.15674900

H -4.36915500 -1.38555500 -0.27573300

H -1.24312000 3.17435200 -0.19611400

H -2.43411800 2.52225200 0.73024500

C 4.63360200 -0.02061300 -0.31413000

H 4.85869600 0.63582400 -1.15635400

H 5.07134000 -1.00702200 -0.47328800

C 3.13873200 -0.11590100 -0.09242600

O 2.66589900 -1.19342100 0.37619100

O 2.43187000 0.90268900 -0.35911100

H 5.08310600 0.41476700 0.58482400

**(c) g-a**

C -2.24503900 2.26870600 -1.01717400

C -1.91912700 1.37509300 0.16270800

O -2.84651700 0.26687600 0.17429600

C -0.80135200 -0.67387100 -0.33683800

O 0.16375900 -1.63037100 0.03118200

C -0.50834300 0.76389500 0.11078300

O 0.07084000 0.79578500 1.40401400

C -2.13164700 -0.95909800 0.34996900

N -2.92654600 -2.03801700 -0.16859700

H -2.06850800 1.73942600 -1.95947300

H -1.61763600 3.16450500 -0.99621700

H -2.02635300 1.92763500 1.10391000

H -0.97549900 -0.67756700 -1.42524100

H 1.04988300 -1.32357400 -0.31397200

H 0.13465200 1.29785200 -0.60042300

H 1.01851900 0.53173800 1.30419700

H -1.95720300 -1.14293800 1.41874700

H -3.29262900 2.58031000 -0.98123800

H -2.64218700 -2.90939200 0.26850900

H -2.77997600 -2.13170300 -1.17174400

C 4.55578800 0.30064800 -0.46476400

H 4.95767700 -0.37674700 -1.21908400

H 5.20062700 0.31200900 0.41588900

C 3.14045800 -0.07941100 -0.08139400

O 2.69966200 0.33135300 1.03095300

O 2.46275500 -0.76550600 -0.90582800

H 4.53604300 1.31370700 -0.88033900

# Supplementary Item References

1. Zhao Y, Truhlar DG. The M06 suite of density functionals for main group thermochemistry, thermochemical kinetics, noncovalent interactions, excited states, and transition elements: two new functionals and systematic testing of four M06-class functionals and 12 other functionals. Theoretical Chemistry Accounts. 2007;120(1-3):215-41. doi: 10.1007/s00214-007-0310-x.

2. Rassolov VA, Ratner MA, Pople JA, Redfern PC, Curtiss LA. 6-31G Basis Set for Third-Row Atoms. Journal_of_Computational_Chemistry. 2001;22:8.

3. Marenich AV, Cramer CJ, Truhlar DG. Universal solvation model based on solute electron density and on a continuum model of the solvent defined by the bulk dielectric constant and atomic surface tensions. J Phys Chem B. 2009;113(18):6378-96. doi: 10.1021/jp810292n. PubMed PMID: 19366259.

4. IUPAC, Compendium of Chemical Terminology, 2nd ed. (the "Gold Book") Online corrected version: (2006–) "endo, exo, syn, anti". 1997.

5. Frisch MJ, Trucks GW, Schlegel HB, Scuseria GE, Robb MA, Cheeseman JR, et al. Gaussian 09, Revision D.01. Gaussian, Inc, Wallingford CT. 2009.

6. Peng C, Ayala PY, Schlegel HB. Using Redundant Internal Coordinates to Optimize Equilibrium Geometries and Transition States. J Comp Chem. 1995;17:7.

7. Cramer CJ, Truhlar DG. Correlation and Solvation Effects on Heterocyclic Equilibria in Aqueous-Solution. Journal of the American Chemical Society. 1993;115(19):8810-7. doi: DOI 10.1021/ja00072a039. PubMed PMID: WOS:A1993LZ13300039.
